# Absolute quantitation of disease protein biomarkers in a single LC-MS acquisition using apolipoprotein F as an example

**DOI:** 10.1038/s41598-017-12229-2

**Published:** 2017-09-21

**Authors:** Abhinav Kumar, Bevin Gangadharan, Jeremy Cobbold, Mark Thursz, Nicole Zitzmann

**Affiliations:** 1 Oxford Antiviral Drug Discovery Unit, Oxford Glycobiology Institute, Department of Biochemistry, University of Oxford, South Parks Road, Oxford, OX1 3QU United Kingdom; 2 Oxford University Hospitals NHS Foundation Trust, John Radcliffe Hospital, Headley Way, Headington, Oxford, OX3 9DU United Kingdom; 3 Division of Digestive Diseases, Imperial College, St Mary’s Hospital Campus, Norfolk Place, London, W2 1NY United Kingdom

## Abstract

LC-MS and immunoassay can detect protein biomarkers. Immunoassays are more commonly used but can potentially be outperformed by LC-MS. These techniques have limitations including the necessity to generate separate calibration curves for each biomarker. We present a rapid mass spectrometry-based assay utilising a universal calibration curve. For the first time we analyse clinical samples using the HeavyPeptide IGNIS kit which establishes a 6-point calibration curve and determines the biomarker concentration in a single LC-MS acquisition. IGNIS was tested using apolipoprotein F (APO-F), a potential biomarker for non-alcoholic fatty liver disease (NAFLD). Human serum and IGNIS prime peptides were digested and the IGNIS assay was used to quantify APO-F in clinical samples. Digestion of IGNIS prime peptides was optimised using trypsin and SMART Digest™. IGNIS was 9 times faster than the conventional LC-MS method for determining the concentration of APO-F in serum. APO-F decreased across NAFLD stages. Inter/intra-day variation and stability post sample preparation for one of the peptides was ≤13% coefficient of variation (CV). SMART Digest™ enabled complete digestion in 30 minutes compared to 24 hours using in-solution trypsin digestion. We have optimised the IGNIS kit to quantify APO-F as a NAFLD biomarker in serum using a single LC-MS acquisition.

## Introduction

Various protein biomarkers in serum and plasma are currently being measured in the clinic using antibody-based approaches. Examples of biomarkers currently analysed by immunoassay include C-reactive protein^1^ and transferrin^2^, which are used to assess inflammation and iron overload, respectively. For each biomarker assay different standards are required to establish calibration curves. We describe an approach which allows up to six different protein biomarkers to be analysed using the same calibration curve which avoids the need for separate biomarker standards and separate calibration curves. To our knowledge this is the only biomarker assay using a universal calibration mix.

Current immunoassays require each point of the standard curve to be read separately, and although this may not always be very time consuming, in a hospital setting samples may be analysed several hours after establishing a calibration curve by which time the instrument drift^3^ may significantly affect accuracy and precision during quantitation of the target molecule. The assay we describe here measures all points on the standard curve and determines the biomarker concentration in the sample in a single acquisition thereby avoiding problems with instrument drift and the need to assay points on a calibration curve separately. This assay allows a 4 to 8 point calibration curve to be established and quantitation of 2 to 6 biomarkers in a single LC-MS acquisition. The assay is up to 9 times faster than the conventional LC-MS based method for protein quantitation and is potentially faster than immunoanalysers.

Unlike existing biomarker immunoassays in the clinic, the assay we describe is antibody-free. Immunoassays require antibodies to recognise a specific sequence on a biomarker. However, if the protein is degraded due to long term storage or freeze/thaw cycles^4^ and the target sequence is no longer intact then the antibody will not detect the biomarker. Our assay will work long after a protein has begun the process of degradation as long as the tryptic peptides of interest are still intact. Another potential difficulty in immunoassays is the generation of biomarker specific antibodies. The MS based assay presented here can be applied to any protein, even if antibodies are unavailable.

We have previously identified apolipoprotein F (APO-F) as a biomarker for liver fibrosis in hepatitis C patients^5^ and developed a multiple reaction monitoring (MRM) method to quantify APO-F^6^. In the study herein we detect and quantify APO-F using parallel reaction monitoring (PRM) where all MS2 ions are observed and so unlike MRM there is no need to preselect fragment ions. PRM provides better selectivity and specificity due to a high resolution and accurate mass (HR/AM)^7^. Targeted quantitation using MRM or PRM is suitable for the clinic and provides better sensitivity, selectivity and reproducibility over shotgun proteomics^8–15^.

**Figure 1:**
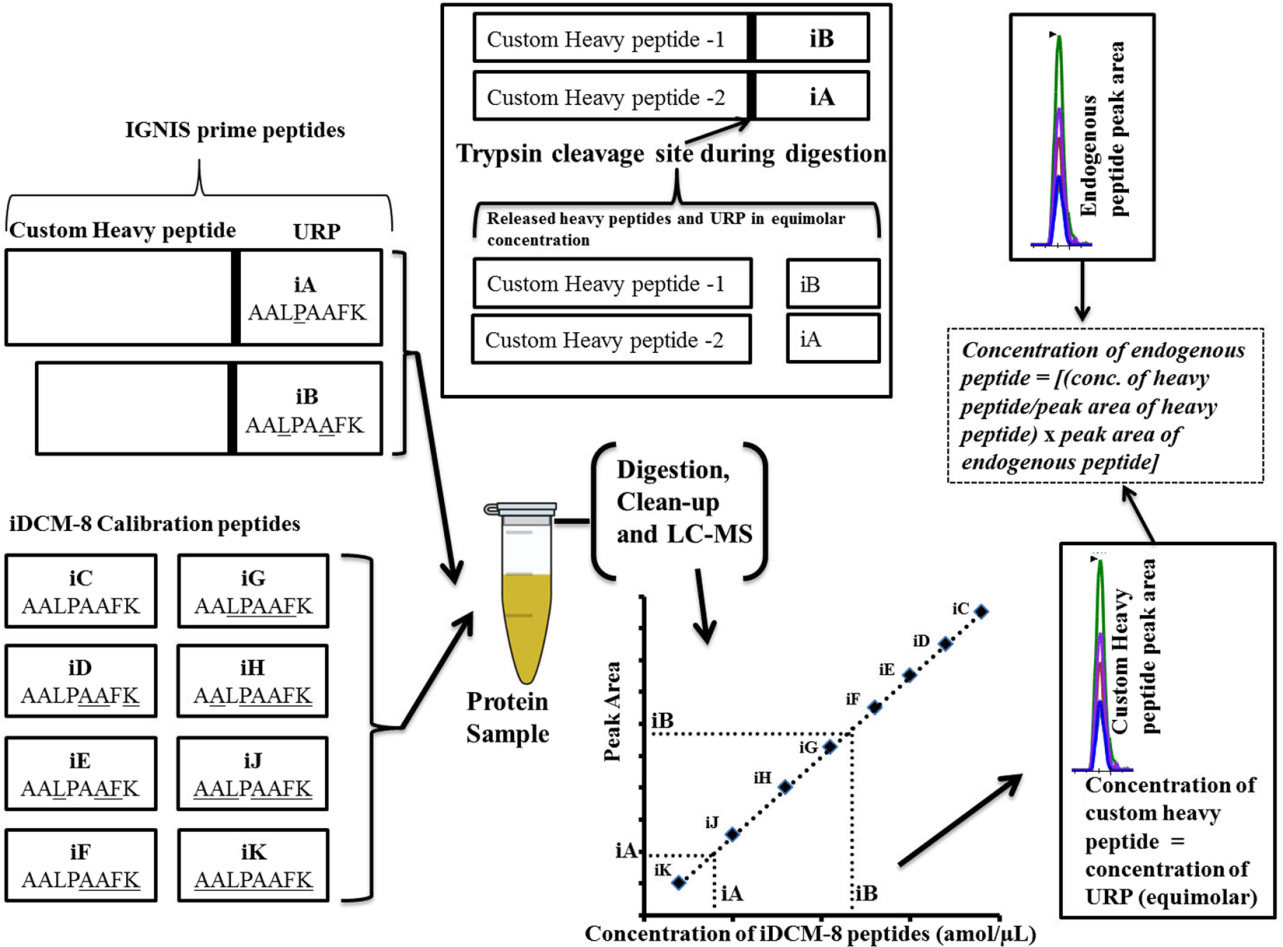
The IGNIS based approach for absolute quantitation of two peptides. The iDCM-8 mixture provides a calibration curve to quantify released URP (iA and iB). IGNIS prime peptides consist of a unique reporter peptide (URP) usually with its N-terminal end attached to a lysine (K) or arginine (R) amino acid at the C-terminus of a custom heavy peptide. The custom heavy peptides and light endogenous peptides have the same peptide sequences (but with different masses), and so their retention time and MS/MS fragmentation patterns are identical. Since URP and the custom heavy peptide are in the same sequence (IGNIS prime peptides), after trypsin digestion they will both be released in equimolar concentration (box in top middle). The absolute concentration of URP can be determined using the iDCM-8 calibration curve and this concentration will be identical to that of the custom heavy peptide. The concentration of custom heavy peptide and its peak area can be used to calculate the concentration of endogenous light peptide by measuring its peak area. The custom heavy peptide and the endogenous light peptide are identical in sequence but can be differentiated by mass spectrometry due to their different mass. Underlined amino acids are isotopically heavy labelled.

The assay we describe is the HeavyPeptide IGNIS Prime Peptide Quantitation kit from Thermo Scientific which uses a cleavable reporter peptide^16^. We compare IGNIS with the conventional LC-MS approach for absolute quantitation using AQUA peptides. Absolute quantitation is the actual concentration of a peptide/protein in a given sample without comparing to another sample which is relative quantitation^17^. In the conventional approach for absolute quantification a multiple point calibration curve is required. Different amounts of AQUA standards were spiked into a digested blank matrix and were run separately by LC-MS followed by the digested sample. The IGNIS approach presented here avoids the need to run separate points of a calibration curve and instead determines all these points and the biomarker concentration in a single acquisition (Fig. 1).

Non-alcoholic fatty liver disease (NAFLD) is the most common liver disorder in the Western world affecting up to 1 in 3 adults^18^. It is a spectrum of progressive liver disease ranging from non-alcoholic fatty liver (NAFL, also known as simple steatosis) to non-alcoholic steatohepatitis (NASH) which can develop with fibrosis and cirrhosis. Accurate diagnosis of disease stage is essential for patient management. Liver biopsy is the current reference standard which is invasive, painful, and only samples 1 in 50,000^th^ of the total liver mass raising the possibility of misclassification especially if steatosis and fibrosis are not homogenous throughout the liver^19^. We decided to use IGNIS to investigate whether APO-F is an informative biomarker for NAFLD because a less invasive and reliable serum biomarker test is required to reduce the need for biopsy. We are working towards the first antibody-free biomarker assay for NAFLD and liver fibrosis. To our knowledge this is the first study using IGNIS for absolute quantitation of a biomarker in clinical samples.

## Results And Discussion

### Digestion of IGNIS prime peptides

The HeavyPeptide IGNIS Prime Peptide quantitation kit was used to quantify APO-F in serum using three preselected peptides. Initially the manufacturer’s recommended protocol was followed where the IGNIS prime peptides and iDCM-8 isotopologues were spiked into a human serum sample and were digested in-solution with trypsin (Methods and Supplementary Methods). When following this protocol undigested IGNIS prime peptides were observed (Supplementary Fig. S1) along with released URP-1 and heavy peptide-1. As complete digestion of the IGNIS prime peptides is required for data analysis the optimum amount of trypsin and time required for complete digestion of IGNIS prime peptides were investigated separately without spiking into serum (Supplementary Methods).

**Figure 2:**
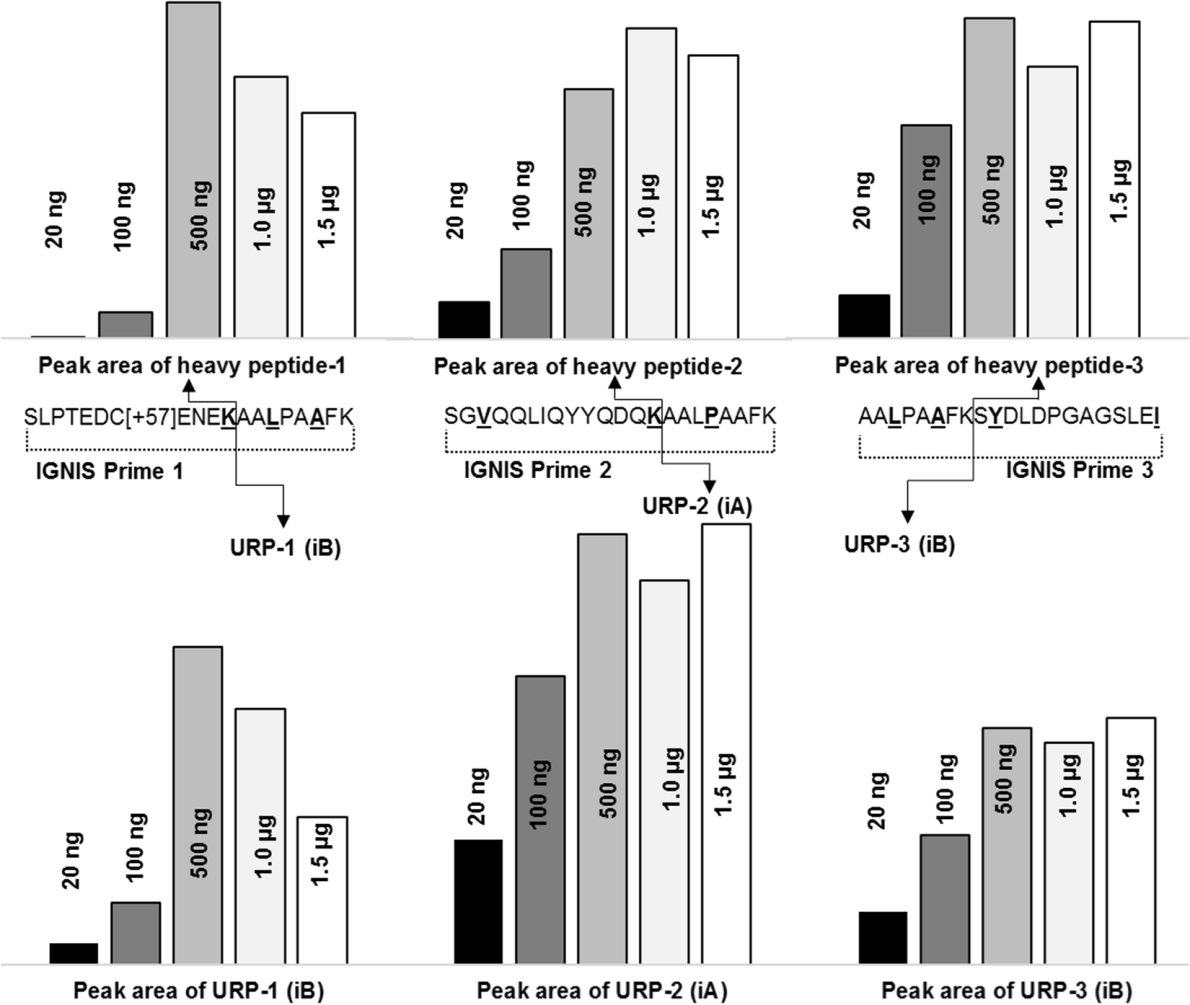
The effect of trypsin amount on the digestion of IGNIS prime peptides 1, 2 and 3. The upper panel shows the peak area of released custom heavy peptides and the lower panel shows the peak area of released URPs from IGNIS prime peptides with varying amounts of trypsin during in-solution digestion. The optimum amount of trypsin for complete digestion was 500 ng for IGNIS prime 1 and 1 µg (between 500 ng to 1.5 µg) for IGNIS prime peptides 2 and 3.

The optimal amount of trypsin was determined using varying concentrations of trypsin (from 20 ng to 1.5 µg in 2 µL volume). After digestion, the samples were analysed separately using PRM targeting the released heavy peptides, URP and undigested IGNIS prime-1. The peak area of released heavy peptide-1 and URP-1 from IGNIS prime-1 increased with increasing amounts of trypsin up to 500 ng followed by a small decrease in the peak area of released peptides with 1 µg and 1.5 µg trypsin (Fig. 2). It was concluded that 500 ng of trypsin was required to completely digest 20 ng of IGNIS prime-1 since this peptide could not be detected when using at least 500 ng trypsin (Supplementary Fig. S2). At least 500 ng of trypsin was also required to digest 20 ng of IGNIS prime-2 and 3. The peak area of both released heavy peptide-2 and 3 and URP-2 and URP-3 were reasonably constant (%CV 5.4 to 10.9%) using 500 ng, 1 μg and 1.5 μg trypsin.

To our knowledge this is the first time an IGNIS prime peptide (IGNIS prime-3) has been synthesised with the URP at the N-terminus of a custom heavy peptide and both trypsin digestion and quantitation have been successful. Although Thermo Fisher state that the selected tryptic peptide should end in a lysine or arginine, we show for the first time that it is possible to use the kit for the C-terminal peptide of a protein which does not end in a lysine or arginine. This shows that the IGNIS kit may also work for other peptides lacking a lysine or arginine as long as they meet the peptide selection criteria. Various trypsin incubation times were used to check IGNIS prime digestion. IGNIS prime-1 was digested with 500 ng trypsin and IGNIS prime-2 and 3 with 1 µg trypsin. Samples were incubated for various times up to 22 hours. Complete digestion for IGNIS prime-1 was observed at four hours where the peak areas for both peptide-1 and URP-1 were highest. For IGNIS prime-2 and 3 complete digestion was between 2 to 3 hours since the peak areas of URP-2 and 3 were highest at 2 hours and the peak areas of peptide-2 and 3 were highest at 3 hours (Supplementary Fig. S3). A decrease in the concentration of released heavy peptides and URPs were observed after these optimal incubation times.

### Detection of APO-F in serum and plasma by PRM

**Figure 3:**
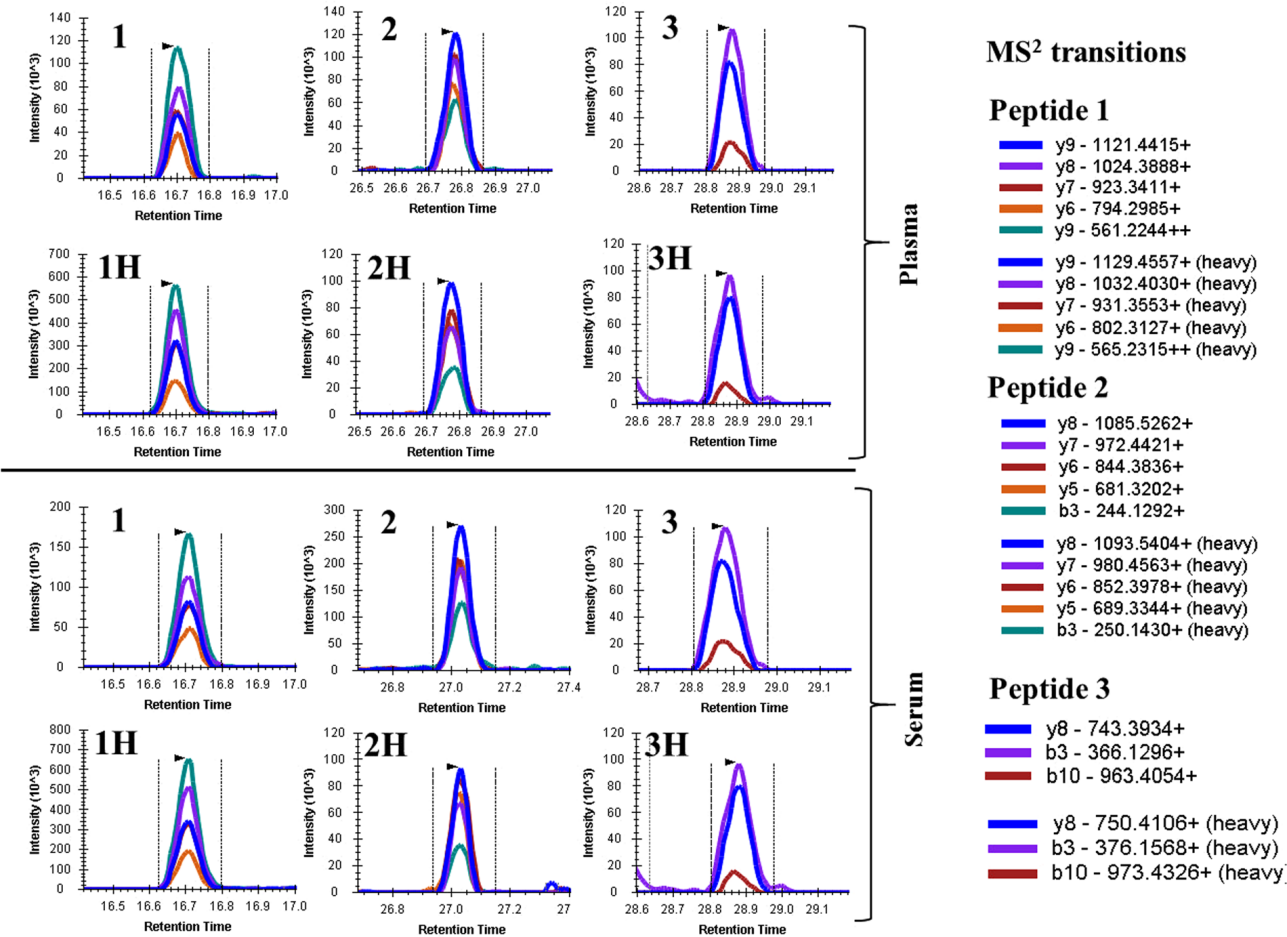
Chromatograms of endogenous and spiked heavy peptides in undepleted and unfractionated human plasma and serum. The upper and lower panels show the chromatograms of peptides in plasma and serum, respectively. (**1**), (**2**) and (**3**) represent the chromatograms of endogenous peptide 1 (SLPTEDC[+57]ENEK), peptide 2 (SGVQQLIQYYQDQK) and peptide 3 (SYDLDPGAGSLEI), respectively. 1H, 2H and 3H represent the corresponding heavy peptides in plasma and serum to confirm the retention times of respective endogenous light peptides. MS^2^ transitions show the lists of y and b ions considered for measuring the peaks of each peptide. The dotp values for peptides (**1**), (**2**) and (**3**) were ≥ 0.95 in both plasma and serum samples.

Three APO-F peptides were targeted in human serum (Fig. 3). We have previously used MRM to detect and quantify APO-F^6^ and found that the peptide DANISQPETTK was unsuitable since it was glycosylated and so was not included for analysis by PRM. Unlike our previous MRM study^6^, we decided to include a cysteine containing peptide SLPTEDC[+57]ENEK since reduction and alkylation steps were used during our digestion protocol. The retention time for each endogenous peptide matches with the retention time of the corresponding spiked heavy peptide. The identical retention time gives confidence in true peak picking for the selected peptides. In order to confirm the identity of endogenous peptide and to rule out interference due to the matrix the chromatographic elution pattern of MS^2^ transition ions and the dot product (dotp) value were also considered. A dotp value closer to 1 indicates better matching of the endogenous peptide peaks with the library. The dotp values for both light and heavy peptides were between 0.94–0.96 and the mass error for the observed MS^2^ ions of all endogenous and heavy peptides were ≤1.2 ppm. The high dotp and low mass error values gives confidence in true peak detection and indicates that all three endogenous peptides are free from interference from the serum and plasma samples. Similarly all spiked heavy labelled peptides were free from interference and so were suitable for absolute quantitation of endogenous peptides.

### Absolute quantitation of APO-F in serum

**Figure 4:**
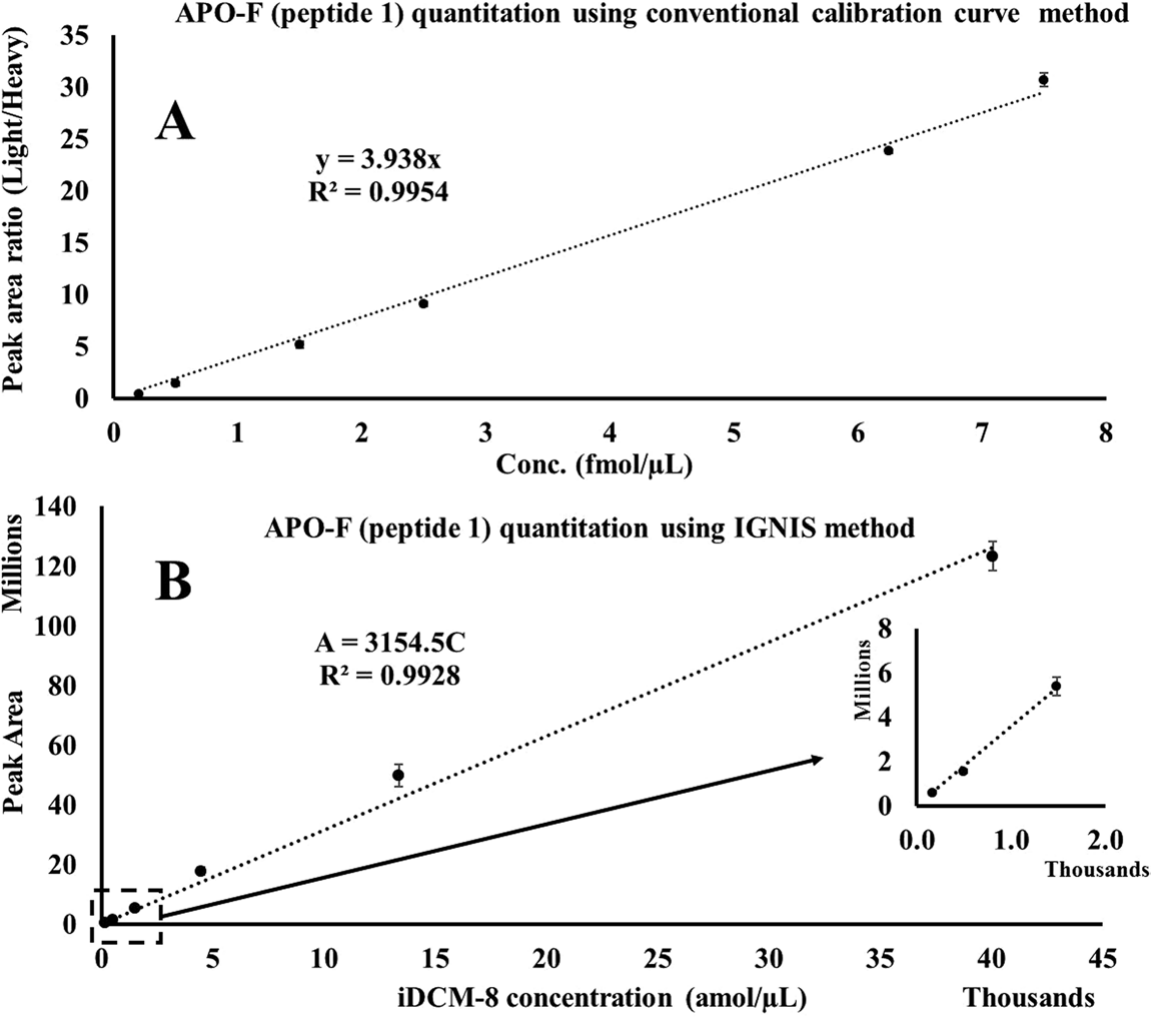
Calibration curves for APO-F. (**A**) Six point calibration curve of peptide 1 in a digest of fetal calf serum (100 ng/µL). The lowest point on the calibration curve (0.2 fmol/µL) was 4 times higher than the limit of detection (LOD, 0.05 fmol/µL, see Supplementary information). A fixed amount of heavy peptide 1 (0.4 fmol/µL) was spiked into varying concentrations of light peptide 1 and the peak area ratios of light peptide 1 to heavy peptide 1 were plotted against the concentrations. Each point on the calibration curve is the average of three repeat injections with %CV varying between 0.7 to 10.9%. Two quality control (QC) samples at 0.3 fmol/µL and 3.75 fmol/µL were used to check the accuracy of quantification (see Supplementary Table 3). (**B**) Six point calibration curve obtained from iDCM-8 using the IGNIS approach. Only six iDCM-8 peptides (iC, iD, iE, iF, iG and iH) out of eight isotopologues were used in this calibration curve. The two isotopologues iJ and iK were not detected due to low concentration and when a high concentration of iDCM-8 was used to try and detect these isotopologues, a carryover problem for the most concentrated isotopologue, iC, was observed. An average of three repeat injections were used to plot the calibration curve (% CV = 3.9 to 11.4, Supplementary Table 4) and the absolute concentration of APO-F in three repeat injections were 148.1, 139.15 and 142.8 amol/100 ng of serum (average = 143.3 amol/100 ng, % CV = 3.11). Error bars added to each point across the calibration curve show the standard deviation.

Using the conventional approach for APO-F quantitation, the equation of the line for the calibration curve was A = 3.938 C, where A = peak area ratio (light/heavy) and C = concentration on column (R^2^ = 0.9954, Fig. 4A, Supplementary Table 3). The percent accuracy of calculated amounts of quality control (QC) samples was 86.3% in the lower region (0.3 fmol/µL) and 87.6% in the middle region (3.75 fmol/µL) of the calibration curve. Quantitation of APO-F in human serum was carried out by spiking the same amount of heavy peptide-1 (0.4fmol/µL) into 100 ng/µL of digested human serum and measuring the ratio of the peak area of the endogenous light peptide 1 to heavy peptide-1. This ratio was determined to be 0.58 and using the calibration curve this gives a concentration for APO-F of 147.7 amol/100 ng of human serum (Supplementary Table 3). We have not used external calibrators in this study but this has been successfully used by Van den Broek and coworkers using a reference material^20^.

Using the IGNIS approach, the same endogenous peptide-1 and released peptides from digested IGNIS prime-1 were targeted. The concentration of APO-F in the same human serum sample was determined to be 143.3 amol/100 ng of serum (average of three repeat injections, % CV = 3.1). The equation of the line for the IGNIS based calibration curve was A = 314.5 C, where A = peak area of IGNIS iDCM-8 peptides and C = concentration of IGNIS iDCM-8 peptides on column (R^2^ = 0.9928, Fig. 4B). The calculation for obtaining the APO-F concentration is shown in Supplementary Table 4. To calculate the concentration of endogenous peptides the IGNIS method uses the equivalent of a one point calibration. Hence, endogenous peptide-1 was detected in different concentrations of human serum and was found to be linear (Supplementary Methods and Supplementary Figure S11).

The %CV of the isotopologues shows a general increase with a decrease in their intensity/concentration (Supplementary Table S4). The isotopologue with the lowest intensity (iH) has the highest %CV (11.39%) and the isotopologue with the highest intensity (iC) has the lowest %CV (3.94%). This could be due to the lower concentration of iH and so there is more ion suppression from the co-eluting high abundant peptides. However, the intensity of light peptide-1 is almost 2 times lower than heavy peptide-1 and their %CVs are the same (8.2%). Also, the average intensity of iB and endogenous peptide-1 are similar but the %CV of iB is lower (3.9%) than endogenous peptide-1 (8.2%). This suggests that the variation in %CV could be peptide sequence specific and is not inherent to the Q Exactive.

Both the conventional and IGNIS approaches for quantitation gave similar concentrations of APO-F in 100 ng of normal human serum with only a 3% difference between the concentration values which validates the use of the new IGNIS approach for absolute quantification of proteins. The IGNIS method, however, is up to 9 times faster than the conventional LC-MS method for protein biomarker quantitation which would be a significant advantage in a clinical test. The conventional LC-MS method to quantify APO-F using peptide-2 and 3 was not carried out due to carryover for these peptides when using the highest concentrations of calibration curve and it was very difficult to remove these peptides from the column. The absolute concentrations of APO-F found when applying the IGNIS method to peptide-2 and 3 were 425.87 amol/100 ng (n = 3, %CV = 13.1) and 306.33 amol/100 ng (n = 3, %CV = 15), respectively. Peptide-2 was previously reported to have a very similar concentration value determined using MRM and a conventional method (13.12 fmol/3 µg plasma digest = 437.3 amol/100 ng plasma digest)^6^. Since all three peptides are released from the same protein their absolute concentration should be equimolar; however the concentrations of peptide-1 and 3 are 2.9 and 1.4 times lower than peptide 2, respectively. This difference could be due to presence of post-translational modifications on peptide-1 and 3. Peptide-1 has a cysteine and it is possible that it has not been completely alkylated. Peptide-3 has a phosphorylation site on the serine amino acid at position 323^21^, as observed when creating a peptide library from a digest of human plasma (Supplementary Fig. S8).

### Precision and reproducibility of the IGNIS method

The intra-day precision (%CV) of three repeat injections of the sample for determining the absolute concentration of APO-F was 3.1%, 13% and 15% using peptides-1, 2 and 3, respectively. The inter-day variations for the three different sample preparations (with three repeat injections for each sample), from the same stock of human serum, were 13.6%, 11.0% and 26.7% for peptide-1, 2 and 3, respectively and for the 6 different stocks the %CV values were very similar (Supplementary Table 5). The %CV variation for peptide-1 and 3 was higher compared to peptide-2 and this could be due to different levels of alkylation and phosphorylation in peptide-1 and 3, respectively.

### Post sample preparation stability

As the reliability of the method will also rely on the stability of peptides after sample preparation, this was checked after spiking iDCM-8 and digested IGNIS prime peptides into a digest of serum. The variation in the absolute concentration of APO-F was then assessed in the same sample over 28 hours at 5 °C. The sample was analysed by a fixed LC-MS acquisition method every four hours and the absolute concentrations of APO-F were compared. The absolute value of APO-F using peptide-1 was constant up to 28 hours (% CV = 6.1%) and using peptide-2 and 3 the values were constant for 24 hours (%CV < 10%) followed by a 17% and 32% decrease at 28 hours with respect to the average value up to 24 hours. This shows that all three peptides can be used for absolute quantitation within 24 hours of sample preparation (Supplementary Fig. S9) and a sample must be analysed within 24 hours of sample preparation when stored at 5 °C.

### Validation of APO-F in NAFLD using clinical samples

**Figure 5:**
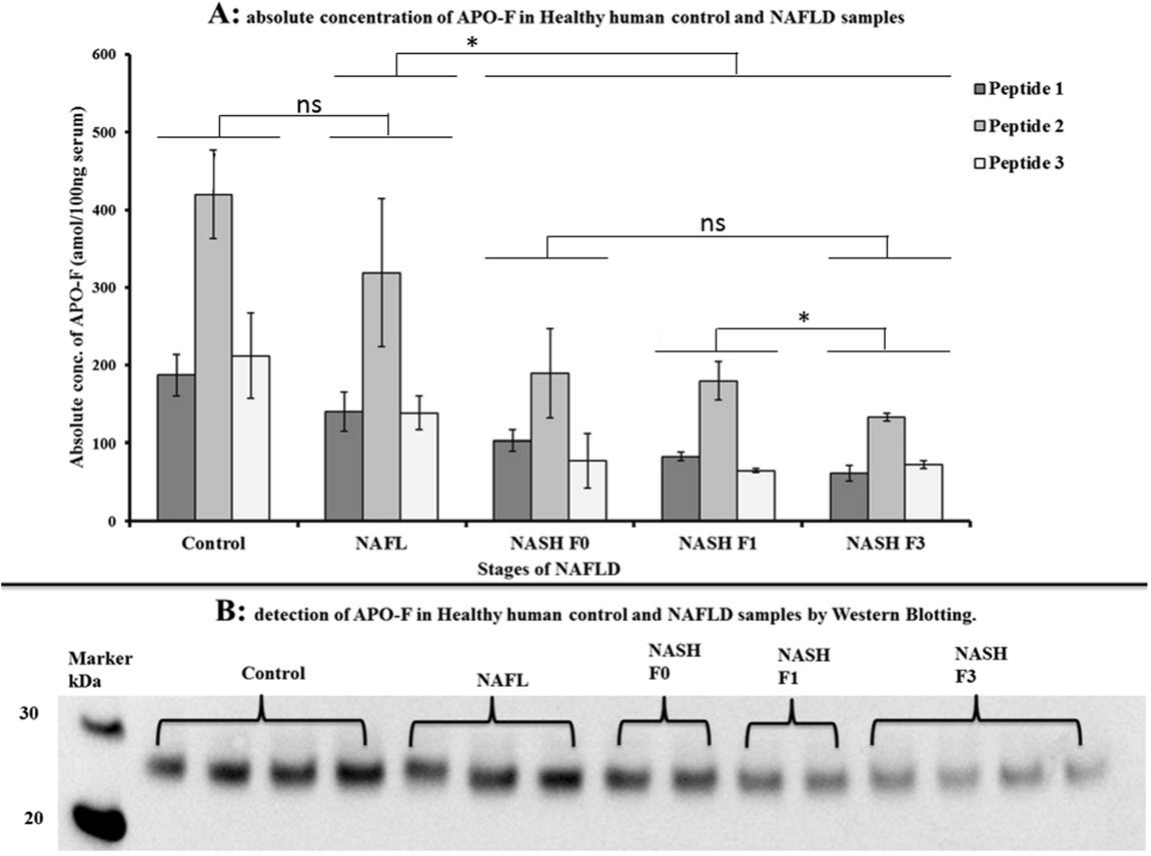
Detection of APO-F in NAFLD clinical samples. (**A**) Absolute concentration of APO-F determined by the IGNIS method using serum samples from patients across different stages of NAFLD. APO-F can clearly distinguish NASH (F3/F1/F0) from healthy control and NAFL (p < 0.05, Supplementary Table 6); and between F3 and F1 stages (using peptide 2). Sample size: Control = 4, NAFL = 3, NASH F0 = 2, NASH F1 = 2, NASH F3 = 4. (**B**) Detection of APO-F in NAFLD samples by western blotting. *Significantly different (p < 0.05), ns - not significant (Supplementary Table 6).

The IGNIS method we developed for APO-F was used to quantify this biomarker in serum samples from NAFLD patients. The concentration of APO-F calculated using all three peptides decreased with the progression of NAFLD and this data is consistent with the Western blot data for the same NAFLD serum samples (Fig. 5). We previously showed that the concentration of APO-F decreases with progression of liver fibrosis in hepatitis C patients^5^. The LC-MS data presented here show a similar correlation in NAFLD. We show that not only does APO-F help to determine fibrosis stage, but it can also differentiate between healthy individuals, patients with NAFL and patients with steatohepatitis. The results presented here demonstrate that APO-F is a general liver disease biomarker and not specific to hepatic fibrosis. Levels of APO-F in serum samples from patients with NASH are clearly different from healthy controls and NAFL (p < 0.05, Supplementary Table 6). There is also a statistically significant difference between fibrosis stages F1 and F3 using peptide 2 (p < 0.05, Supplementary Table 6). Hence using the IGNIS method we can absolutely quantify APO-F in human serum which can be used to help determine the stage of NAFLD.

### SMART digest™ digestion of IGNIS prime

Although the levels of APO-F could be quantified using a standard trypsin digest, this is a slow step in the protocol, and in-solution trypsin digestion requires a very high trypsin concentration to achieve complete digestion of synthetic IGNIS prime peptides. The SMART Digest™ kit offers a more rapid digestion process, therefore we tested whether this kit was appropriate for protein quantitation using the IGNIS method. The SMART Digest™ trypsin digestion kit provided a complete digestion of IGNIS prime-1 and human serum in just 30 mins (see Supplementary results and Figures S4 and S6). By using this digestion kit with the IGNIS method, separate in-solution digestion of IGNIS prime and human serum samples can be avoided leading to an overall faster analysis.

## Conclusion

We have optimised the novel IGNIS quantitation kit which is the only method capable of establishing a universal calibration curve and determining the concentration of a disease biomarker in a single run. IGNIS was found to be up to 9 times faster than the conventional approach of protein quantitation by mass spectrometry when using the same digested sample and identical LC method.

We have for the first time developed an IGNIS method for APO-F and have preliminary data to show that this is a novel biomarker for NAFLD. We plan to use a large cohort of NAFLD samples to validate APO-F as a NAFLD biomarker and hope this assay may help reduce the need for invasive liver biopsies. We have also shown that the SMART Digest™ kit significantly reduces digestion time for human serum and synthetic peptides which can be implemented in the IGNIS quantitation work flow. IGNIS combined with SMART Digest^TM^ kit can produce absolute quantitation data in 1.5 hours from the time of receiving a clinical sample. Conventional quantitation with SMART Digest^TM^ kit would still take up to 9 times longer (using the same LC method) to obtain absolute quantitation data and this is due to separate LC-MS acquisitions for each point on the calibration curve, each quality control standard and the clinical sample.

## Materials and Methods

### NAFLD samples and ethical approval

The IGNIS kit was used to determine the absolute concentration of APO-F in serum samples from patients with various stages of NAFLD. 4 control serum samples were collected from healthy volunteers at the John Radcliffe hospital, Oxford, UK. 11 serum samples from NAFLD patients were obtained from hospitals within the Imperial College Healthcare NHS Trust, London, UK (Supplementary Table 2). Ethical approval was given by the West London Research Ethics Committee; reference number 10/H0711/58 and informed consent was obtained from all subjects. See Supplementary Methods and Supplementary Table 2. All experiments were performed in accordance with relevant guidelines and regulations. All the experiments on control and NAFLD samples were approved by Department of Biochemistry, University of Oxford, UK.

## Methods

See the Supplemental Data section for the methods used for LC, Skyline (the software used for *in-silico* protein digestion and data processing), sample preparation, reference library construction, IGNIS/conventional absolute quantitation of biomarkers and western blotting. A benchtop Q Exactive hybrid quadrupole-Orbitrap mass spectrometer and a Dionex Ultimate 3000 nanoLC system (Thermo Scientific) was used for data acquisition using parallel reaction monitoring (PRM) and data dependent acquisition (DDA, see supplementary method for more information). MS settings for PRM acquisition were: global settings – user role: advanced; lock mass: best; chromatographic peak width: 15 s; t-MS2 settings – polarity: positive; in-source collision induced dissociation (CID): 0.0 eV; default charge: 2; inclusion: on; microscan: 1; resolution: 35000; AGC target: 3e6; Maximum injection time (IT): 100 ms; MSX count: 1; isolation window: 1.0 m/z; normalised collision energy (NCE): 27; spectrum type: profile. The MS tune file for nano flow rate at 300 nL/min was used with following settings: scan type: full MS-SIM, scan range: 300–2000 m/z, fragmentation: none, resolution: 70000, polarity: positive, microscan: 1, AGC target: 1e6, maximum IT: 100, sheath gas flow: 0, aux gas flow: 0, sweep gas flow: 0, spray voltage: 2.3 kV for peptide 1 and 2.0 kV for peptide 2 and 3, capillary temperature: 320 °C, S-lens RF level: 55. Inclusion list contains precursor ions of light and heavy labelled (underlined amino acids) peptides AALPAAFK (m/z = 394.737, +2), AALPAAFK (m/z = 397.744, +2, **iA URP**), AALPAAFK (m/z = 400.249, +2, **iB**, **URP**), AALPAAFK (m/z = 402.751, +2), AALPAAFK (m/z = 405.263, +2), AALPAAFK (m/z = 407.765, +2), AALPAAFK (m/z = 410.273, +2), AALPAAFK (m/z = 412.775, +2), AALPAAFK (m/z = 415.280, +2), AALPAAFK (m/z = 418.287, +2), SLPTEDC[+57]ENEK (peptide 1, m/z = 661.283, +2), SGVQQLIQYYQDQK (peptide 2, m/z = 849.428, +2), SYDLDPGAGSLEI (peptide 3, m/z = 668.817, +2), SLPTEDC[+57]ENEK (heavy peptide 1, m/z = 665.289, +2), SGVQQLIQYYQDQK (heavy peptide 2, m/z = 856.442, +2), SYDLDPGAGSLEI (heavy peptide 3, m/z = 677.339, +2).

## Materials

Solvents used for LC-MS analysis were of mass spectrometry (MS) grade. Acetonitrile, HPLC water, formic acid and trifluoroacetic acid (TFA) were obtained from Thermo Fisher (UK). Sequencing grade modified trypsin was purchased from Promega (UK). All AQUA peptides (light: SLPTEDC[+57]ENEK and heavy: SLPTEDC[+57]ENEK, SGVQQLIQYYQDQK and SYDLDPGAGSLEI; underlined amino acids indicate that they have a heavy stable isotope C^13^/N^15^ label) and IGNIS peptide quantitation kits for these three target peptides were purchased from Thermo Scientific GmbH (Germany). The SMART Digest™ trypsin kit was purchased from Thermo Scientific (UK).

### HeavyPeptide IGNIS Prime Peptide Quantitation

The IGNIS quantitation kit was marketed by Thermo Scientific. We describe the IGNIS approach in detail below since the manufacturer’s method is complex and is currently not published nor available online. The IGNIS quantitation kit is based on the principle described by Duriez *et al*.^16^. It utilises a mixture of isotopologues of the unique peptide AALPAAFK, differing in mass due to heavy isotope labelled amino acids at different positions in its sequence (Table 1, Supplementary Table 1). Isotopologues, which have more than 95% purity, can be included at different concentrations to generate a universal calibration curve. The precursor m/z ions for these unique isotopologue peptides are targeted by mass spectrometry. The isotopologues co-elute at the same retention time due to their identical sequences. The protocol requires the selection of unique reporter peptides (URPs); only isotopologues iA, iB, iC, iD, iE and iF (Table 1) can be selected as a URP. The selected URP is joined at a lysine or arginine at the C-terminus of custom heavy peptide (Fig. 1). The whole sequence (custom heavy peptide + URP) is called an IGNIS prime peptide. We have used three IGNIS prime peptides which are as follows: SLPTEDC[+57]ENEK (custom heavy peptide 1) + AALPAAFK (iB) = SLPTEDC[+57]ENEKAALPAAFK (IGNIS prime-1), SGVQQLIQYYQDQK (custom heavy peptide 2) + AALPAAFK (iA) = SGVQQLIQYYQDQKAALPAAFK (IGNIS prime-2) and AALPAAFK (iA) + SYDLDPGAGSLEI (custom heavy peptide 3) = AALPAAFKSYDLDPGAGSLEI (IGNIS prime-3). The custom heavy peptide should be 7 to 25 amino acids in length and is selected from the sequence of the protein of interest. It must not contain methionine since this amino acid may or may not be oxidised which would change the peptide mass. The dilution curve mixture iDCM-8 contains isotopologues iC, iD, iE, iF, iG, iH, iJ and iK at different concentrations enabling quantitation of two peptides using iA and iB as URPs. A so-called isotopologue bracketing mixture (iBM-4) contains iG, iH, iJ and iK which enables quantitation of six peptides at once (using iA, iB, iC, iD, iE and iF as URPs). After trypsin digestion the custom heavy peptide and URP are released in a 1:1 stoichiometry. Figure 1 shows how absolute quantitation of two target peptides is achieved using IGNIS. We used iA and iB as URPs to quantify two APO-F peptides in a single injection using iDCM-8. iB was used as the URP for the endogenous peptide SLPTEDC[ + 57]ENEK (peptide-1) and iA was used as the URP for endogenous peptides SGVQQLIQYYQDQK and SYDLDPGAGSLEI (peptides-2 and -3, respectively).

**Table 1:** List of IGNIS peptides showing a shift in mass due to isotopically heavy labelled amino acids at different positions of the same unique peptide sequence. Underlined amino acids are isotopically heavy labelled. iA-iF can be selected as unique reporter peptides (URP) for quantitation of 6 biomarkers using a 4 point calibration curve. There are total of 10 isotopologues therefore the quantitation of biomarkers using 2, 3 or 4 URPs would result in a 8, 7 or 6 point calibration curve, respectively.

Isotopologue name	Isotopologue sequence	M (g/mol)	∆m vs. iC	m/z (+2)	Fragment ions type and MS^2^ transitions (m/z)
iC	AALPAAFK	787.459	0	394.737	y6–646.3923+, y5–533.3082+, y5–267.1577++
iA	AALPAAFK (URP for peptides 2 and 3)	793.473	6	397.744	y6–652.4061+, y5–539.3220+, y5–270.1646++
iB	AALPAAFK (URP for peptide 1)	798.483	11	400.249	y6–657.4165+, y5–537.3153+, y5–269.1613++
iD	AALPAAFK	803.488	16	402.751	y6–662.4207+, y5–549.3366+, y5–275.1719++
iE	AALPAAFK	808.511	21	405.263	y6–667.4438+, y5–547.3425+, y5–274.1749++
iF	AALPAAFK	813.515	26	407.765	y6–672.4479+, y5–559.3638+, y5–280.1856++
iG	AALPAAFK	818.532	31	410.273	y6–677.4647+, y5–557.3634+, y5–279.1854++
iH	AALPAAFK	823.536	36	412.775	y6–678.4617+, y5–565.3776+, y5–283.1925++
iJ	AALPAAFK	828.546	41	415.280	y6–679.4651+, y5–559.3638+, y5–280.1856++
iK	AALPAAFK	834.560	47	418.287	y6–685.4789+, y5–565.3776+, y5–283.1925++

Thermo Fisher recommends that the IGNIS prime peptide should be synthesised with the selected URP at the C-terminus of the custom heavy peptide where the custom heavy peptide ends in a lysine (K) or arginine (R) to allow trypsin cleavage. However, peptide 3 (SYDLDPGAGSLEI) is the peptide at the C-terminal end of APO-F and does not end in lysine or arginine. We decided to test a new approach where the IGNIS prime peptide is synthesised with the URP at the N-terminus of the custom heavy peptide. The URP sequence AALPAAFK ends in a lysine which would allow trypsin cleavage.

The peptides used in our earlier study^6^ to quantify APO-F were SGVQQLIQYYQDQK and SYDLDPGAGSLEI (peptides-2 and -3, respectively). In addition to these two peptides, the cysteine containing peptide SLPTEDC[+57]ENEK was also included in this study (peptide-1, Supplementary Figure S7). The three IGNIS prime peptides used for quantification of target custom heavy peptide-1 (SLPTEDC[+57]ENEK), peptide-2 (SGVQQLIQYYQDQK) and peptide-3 (SYDLDPGAGSLEI) were SLPTEDC[+57]ENEKAALPAAFK (IGNIS prime-1), SGVQQLIQYYQDQKAALPAAFK (IGNIS prime-2) and AALPAAFKSYDLDPGAGSLEI (IGNIS prime-3), respectively.

### Trypsin digestion using SMART Digest™

IGNIS prime-1 was spiked into serum from a healthy individual and digested using various incubation times with the SMART Digest™ kit. The digested sample was analysed by LC-MS before and after reduction/alkylation (Supporting Methods).

## Electronic supplementary material

Supplementary information

## References

[1] Rifai N, Ridker PM (2001). High-sensitivity C-reactive protein: a novel and promising marker of coronary heart disease. Clin Chem.

[2] Elsayed ME, Sharif MU, Stack AG (2016). Transferrin Saturation: A Body Iron Biomarker. Adv Clin Chem.

[3] Ramamohan V, Abbott JT, Klee GG, Yih Y (2015). Modeling the effect of instrument drift in clinical laboratories: A serum bilirubin assay case study. IIE Transactions on Healthcare Systems Engineering.

[4] Mitchell BL, Yasui Y, Li CI, Fitzpatrick AL, Lampe PD (2005). Impact of freeze-thaw cycles and storage time on plasma samples used in mass spectrometry based biomarker discovery projects. Cancer Inform.

[5] Gangadharan B (2012). Discovery of novel biomarker candidates for liver fibrosis in hepatitis C patients: a preliminary study. PLoS One.

[6] Kumar A, Gangadharan B, Zitzmann N (2016). Multiple reaction monitoring and multiple reaction monitoring cubed based assays for the quantitation of apolipoprotein F. J Chromatogr B Analyt Technol Biomed Life Sci.

[7] Peterson AC, Russell JD, Bailey DJ, Westphall MS, Coon JJ (2012). Parallel reaction monitoring for high resolution and high mass accuracy quantitative, targeted proteomics. Mol Cell Proteomics.

[8] Parker CE, Pearson TW, Anderson NL, Borchers CH (2010). Mass-spectrometry-based clinical proteomics–a review and prospective. Analyst.

[9] Kirkpatrick DS, Gerber SA, Gygi SP (2005). The absolute quantification strategy: a general procedure for the quantification of proteins and post-translational modifications. Methods.

[10] Phanstiel D, Unwin R, McAlister GC, Coon JJ (2009). Peptide quantification using 8-plex isobaric tags and electron transfer dissociation tandem mass spectrometry. Anal Chem.

[11] Grossmann J (2010). Implementation and evaluation of relative and absolute quantification in shotgun proteomics with label-free methods. J Proteomics.

[12] Matallana-Surget S, Leroy B, Wattiez R (2010). Shotgun proteomics: concept, key points and data mining. Expert Rev Proteomics.

[13] Liu H, Sadygov RG, Yates JR (2004). A model for random sampling and estimation of relative protein abundance in shotgun proteomics. Anal Chem.

[14] Tabb DL (2010). Repeatability and reproducibility in proteomic identifications by liquid chromatography-tandem mass spectrometry. J Proteome Res.

[15] Addona TA (2009). Multi-site assessment of the precision and reproducibility of multiple reaction monitoring-based measurements of proteins in plasma. Nat Biotechnol.

[16] Duriez E, Trevisiol S, Domon B (2015). Protein quantification using a cleavable reporter peptide. J Proteome Res.

[17] Ludwig C, Claassen M, Schmidt A, Aebersold R (2012). Estimation of absolute protein quantities of unlabeled samples by selected reaction monitoring mass spectrometry. Mol Cell Proteomics.

[18] Alkhouri N, McCullough AJ (2012). Noninvasive Diagnosis of NASH and Liver Fibrosis Within the Spectrum of NAFLD. Gastroenterol Hepatol (N Y).

[19] Machado MV, Cortez-Pinto H (2013). Non-invasive diagnosis of non-alcoholic fatty liver disease. A critical appraisal. J Hepatol.

[20] van den Broek I (2016). Automated Multiplex LC-MS/MS Assay for Quantifying Serum Apolipoproteins A-I, B, C-I, C-II, C-III, and E with Qualitative Apolipoprotein E Phenotyping. Clin Chem.

[21] Bian Y (2014). An enzyme assisted RP-RPLC approach for in-depth analysis of human liver phosphoproteome. J Proteomics.

