# Supplementary material for: Absolute quantitation of disease protein biomarkers in a single LC-MS acquisition using apolipoprotein F as an example
Source: Sci Rep. 2017 Sep 21;7:12072. doi: 10.1038/s41598-017-12229-2 (PMC5608892; doi:10.1038/s41598-017-12229-2)
Supplement: Supplementary file 1 — Supplementary information [file 41598_2017_12229_MOESM1_ESM.pdf]

Absolute quantitation of disease protein biomarkers in a single LC-MS acquisition using apolipoprotein F as an example

Abhinav Kumar[1*], *Bevin Gangadharan*[1], *Jeremy Cobbold*[2], *Mark Thursz*[3] *and Nicole Zitzmann*[1]

[1]Oxford Antiviral Drug Discovery Unit, Oxford Glycobiology Institute, Department of Biochemistry, University of Oxford, South Parks Road, Oxford, OX1 3QU, United Kingdom.

[2]Oxford University Hospitals NHS Foundation Trust, John Radcliffe Hospital, Headley Way, Headington, Oxford, OX3 9DU, United Kingdom.

[3]Division of Digestive Diseases, Imperial College, St Mary's Hospital Campus, Norfolk Place, London, W2 1NY, United Kingdom.

*Corresponding Author: abhinav.kumar@bioch.ox.ac.uk (A. Kumar)

Supplementary Methods and Results

Supporting methods

Skyline: Skyline software (version 3.5.0.9319, 64-bit, Seattle, USA) was used for peptide quantitation. The Skyline settings used were as described by Kumar *et al.*, 2016 (1). In-silico digestion for APO-F was achieved by using the following parameters in 'Peptide Settings' (Settings > Peptide Settings) and these settings can be used for in-silico digestion of any protein:- Enzyme: trypsin; peptide length: 7 to 25 amino acids (aa) long (peptide sequences less than 7 aa are not very selective or unique for the protein of interest and peptides with more than 25 amino acids would be more difficult and expensive to synthesize); missed cleavages: none; modifications to be excluded: cysteine and methionine containing peptides as they are potential modification sites for alkylation and oxidation, respectively. A cysteine containing peptide was included (peptide-1) as reduction and alkylation steps are included before digestion with trypsin in order to break any disulfide bonds and protect the resulting free cysteine residues from further modification (assuming that complete reduction/alkylation is achieved); other modifications such as heavy labelling, isotope modifications: none. For targeted quantitation by PRM, the acquisition method in Skyline was changed to 'Targeted' and the product mass analyser to 'Orbitrap' (Skyline > Settings > Transition Settings > Full-Scan).

Liquid chromatography (LC): The nano LC system Dionex Ultimate 3000 (RSLC nano system) was used to separate peptides. A nano analytical C18 reversed phase column (PepMap) was used with dimensions 75 µm x 25 cm, 2 µm particle size (Thermo Scientific) with a flow rate of 300 nL/min at ambient room temperature 24 ^{o}C (± 2 ^{o}C). The mobile phase used was solvent A: 0.1% v/v formic acid in water, and solvent B: 0.1% v/v formic acid in acetonitrile. The gradient used to separate peptides on the analytical column was: 4%

B (0-4 min), 4-44% B (4-33 min), 44-90% B (33-34 min), 90% B (34-44 min), 90-4% B (44-45 min) and 4% B for 15 min (45 – 60 min) for column equilibration.

Mass spectrometry (MS): A bench top Q Exactive hybrid quadrupole-Orbitrap mass spectrometer (Thermo Scientific) was used for data acquisition using parallel reaction monitoring (PRM). MS settings for PRM acquisition were: global settings – user role: advanced; lock mass: best; chromatographic peak width: 15 s; t-MS^2 settings – polarity: positive; in-source collision induced dissociation (CID): 0.0 eV; default charge: 2; inclusion: on; microscan: 1; resolution: 35000; AGC target: 3e^6; Maximum injection time (IT): 100 ms; MSX count: 1; isolation window: 1.0 m/z; normalised collision energy (NCE): 27; spectrum type: profile. The MS tune file for nano flow rate at 300 nL/min was used with following settings: scan type: full MS-SIM, scan range: 300-2000 m/z, fragmentation: none, resolution: 70000, polarity: positive, microscan: 1, AGC target: 1e^6, maximum IT: 100, sheath gas flow: 0, aux gas flow: 0, sweep gas flow: 0, spray voltage: 2.3 kV for peptide 1 and 2.0 kV for peptide 2 and 3, capillary temperature: 320 ^{o}C, S-lens RF level: 55. Inclusion list contains precursor ions of light and heavy labelled (underlined amino acids) peptides AALPAAFK (m/z = 394.737, +2), AALPAAFK (m/z = 397.744, +2, **iA URP**), AALPAAFK (m/z = 400.249, +2, **iB, URP**), AALPAAFK (m/z = 402.751, +2), AALPAAFK (m/z = 405.263, +2), AALPAAFK (m/z = 407.765, +2), AALPAAFK (m/z = 410.273, +2), AALPAAFK (m/z = 412.775, +2), AALPAAFK (m/z = 415.280, +2), AALPAAFK (m/z = 418.287, +2), SLPTEDC[+57]ENEK (peptide 1, m/z = 661.283, +2), SGVQQLIQYYQDQK (peptide 2, m/z = 849.428, +2), SYDLDPGAGSLEI (peptide 3, m/z = 668.817, +2), SLPTEDC[+57]ENEK (heavy peptide 1, m/z = 665.289, +2), SGVQQLIQYYQDQK (heavy peptide 2, m/z = 856.442, +2), SYDLDPGAGSLEI (heavy peptide 3, m/z = 677.339, +2).

Top10 data dependent acquisition (DDA) method settings: A bench top Q Exactive mass spectrometer (Thermo Scientific) was used for data acquisition using the Top10 DDA method. Prior to data acquisition, the mass spectrometer was calibrated for mass accuracy using a positive ion calibration mixture (Thermo Scientific). The conditions for the DDA mode were; Chromatographic peak width: 10 s, the Full MS conditions used- resolution: 70,000, AGC target: 1e6, maximum IT (injection time): 100 ms, scan range: 300 to 2000 m/z. The dd-MS2 conditions - resolution: 17,500. The AGC target conditions- 5e4, maximum IT: 100 ms, loop count: 10 (i.e. Top 10), isolation width: 1.6 m/z, fixed first mass: 120.0 m/z, and the data dependent (dd) settings -under fill ratio: 10% (uses a minimum intensity threshold of 5e4 ions), charge exclusion: unassigned, 1, 8, >8, peptide match; preferred, dynamic exclusion: 30 s. Normalised Collison Energy (NCE) of 27 was used for fragmentation of peptides in a high-energy collision dissociation (HCD) cell. This method allows the selection, fragmentation and detection of ten precursors in a duty cycle time of 1.42 s.

Human serum and fetal calf serum (FCS) sample preparation: All human and calf serum samples were digested using the in-solution trypsin digestion protocol described by Kumar *et al.*, 2016 (1). FCS was used as a blank matrix since it lacks APO-F but is similar in complexity. 10 µL of human plasma or FCS (approximately 600 µg of total protein) was denatured using 50 µL of 8 M urea for 45 min. The denatured proteins were then subjected to disulfide bond reduction by adding 50 µL of 100 mM DTT in 50 mM AMBIC and shaking for 30 min at 56 ^{o}C. The solution was allowed to cool to room temperature for 10 min and 100 µL of 100 mM IAA solution was added. The tube was vortex mixed and kept in the dark for 30 min to allow alkylation to occur. The concentration of urea was reduced to 1 M (to prevent denaturation of trypsin) by adding 200 µL of 50 mM AMBIC which also reduced the concentrations of DTT and IAA to 12.5 mM and 25 mM, respectively. 15 µL (1 µg/µL) of trypsin was added, the solution was mixed well and incubated overnight at 37 ^{o}C. An equal

amount of trypsin was added and the solution was incubated for a further 8 hours at 37 ^{o}C. To inactivate trypsin, 0.5 µL of 100% formic acid or trifluoroacetic acid (TFA) was added and the digested sample was desalted by online desalting procedure.

The IGNIS prime peptides were mixed with the iDCM-8 mixture and spiked into the sample prior to digestion with trypsin (Supplemental Methods). After digestion the sample contained released heavy custom peptides, URPs (iA and iB), iDCM-8 and digested serum containing the target endogenous light peptide. This digested sample was analysed by LC-MS. The precursor ions (MS^1) of each peptide were targeted using PRM in a single LC-MS/MS acquisition. iDCM-8 was used to establish a calibration curve which enabled quantification of iA and iB. The concentrations of the released custom peptides were known as the URP and custom heavy peptide are equimolar. Since the sequences of the custom heavy peptide and endogenous target peptide are identical they have the identical LC retention time and MS fragmentation pattern. The endogenous peptide concentration can be calculated from the relative peak areas of the custom heavy peptide and endogenous peptide. Carryover of the most concentrated isotopologue, iC, was observed on the LC column. To avoid this carryover the iDCM-8 mixture was diluted which resulted in lower intensity and poor detection of both iJ and iK (the least concentrated isotopologues in the iDCM-8 mixture). Isotopologues iJ and iK were therefore not included in the final analysis, resulting in a 6 point calibration curve.

As per the IGNIS manufacturer's recommended protocol, human serum samples (5 µL, 60 µg/ µL) were spiked with the undigested IGNIS prime peptides (5 µL, 10 ng/ µL) and iDCM-8 followed by in-solution trypsin digestion as mentioned above using 15 µL (1 µg/ µL) total trypsin. In addition, due to incomplete digestion of IGNIS prime peptides following recommended protocol above, we also pre-digested the IGNIS prime peptides with trypsin separately (see IGNIS sample preparation section below for more information) before spiking into the sample. After digestion, human serum samples (see below IGNIS sample preparation

section) were spiked with digested IGNIS prime and the iDCM-8 mixture for absolute quantitation of APO-F.

Reference library for APO-F peptides for PRM detection: In the study herein we used three peptides SLPTEDC[+57]ENEK (peptide 1), SGVQQLIQYYQDQK (peptide 2) and SYDLDPGAGSLEI (peptide 3) for PRM. The reference library used for peptides 2 and 3 were the same as described in our previous publication (1) and the reference library for peptide SLPTEDC[+57]ENEK (peptide 1, Supplementary Figure S7) was created on Skyline by spiking synthetic light peptide in a digest of control human serum and acquiring data in data dependent mode on a Q Exactive mass spectrometer.

Conventional way of absolute quantitation of APO-F in human serum: Absolute quantitation of APO-F in unfractionated normal human serum was performed using both the conventional and IGNIS approaches. Using the conventional approach a six point calibration curve was obtained by spiking light peptide 1 in different amounts (0.2, 0.5, 1.5, 2.5, 6.25 and 7.5 fmol/ µL) and a fixed concentration (0.4 fmol/µL) of heavy peptide 1 into 100 ng/µL of digested calf serum and plotting the concentration vs peak area ratio (light/heavy). The lowest point in the calibration curve was 4 times higher concentration than the limit of detection (LOD, 0.05 fmol/µL). Two quality control samples, at the lower (0.3 fmol/µL) and middle (3.75 fmol/µL) concentration region of the calibration curve, were used to check the accuracy of quantification. 1 µL of each sample was injected for LC-MS data acquisition.

IGNIS sample preparation: iDCM-8 mixtures in each vial were dissolved in 300 µL of reconstitution solvent and IGNIS prime in each vial (1 µg per vial) was dissolved with 100

µL of reconstitution solvent. As per manufacture's protocol the concentration of iDCM-8 isotopologues in 300 µL volume are calculated in Supplementary Table 4. After dissolving IGNIS prime to a final concentration of 10 ng/µL, 2 µL was added to 46 µL of 25 mM ammonium bicarbonate for digestion with 2 µL of trypsin (concentration ranges 20, 100, 500, 1000 and 1500 ng). These varying concentrations of trypsin were used to determine the optimal conditions for complete digestion of IGNIS prime peptides. Using the optimal trypsin concentrations to digest IGNIS prime peptides 1, 2 and 3, a time course experiment was carried out to find the time required for complete digestion of these IGNIS prime peptides. The digestion times investigated were 1, 2, 3, 4, 5, 9 and 22 hours and both the released custom heavy peptide and URP were analysed.

After complete digestions of IGNIS prime peptides-1, 2 and 3, they were further diluted with 0.05% v/v TFA before spiking into a digest of serum. IGNIS prime peptide-1 was diluted 100 times and IGNIS prime peptides-2 and 3 were diluted 6 times with 0.05% v/v TFA. Spiking of digested serum was carried out as follows: 2 µL of 100 fold diluted and digested IGNIS prime peptide-1 + 5.5 µL iDCM-8 mixture + 2 µL serum (750 ng/µL) + 5.5 µL 0.05% TFA = 15 µL total volume. For IGNIS prime peptides-2 and 3, digested serum was spiked as follows: 2 µL each of 6 fold diluted and digested IGNIS prime peptide-2 & 3 (i.e 4 µL in total) + 5.5 µL iDCM-8 mixture + 2 µL serum (750 ng/µL) + 3.5 µL 0.05% v/v TFA = 15 µL total volume. 1 µL of this sample was injected for LC-MS analysis. 15 µL total volume contains 100 ng/µL of digested human serum protein and different concentrations of iDCM-8 (see Supplementary Table 1 for details).

Trypsin digestion using the SMART Digest™ kit: 5 µL of IGNIS prime peptide-1 (10 ng/ µL) was spiked into 5 µL of neat human serum from a healthy individual and the mixture was diluted up to 50 µL with water. 150 µL of digestion buffer (SMART digestion buffer) was added to this diluted spiked serum sample and mixed. This sample in digestion buffer was

transferred to a tube containing SMART Digest™ immobilised trypsin and was heated to 70 ^{o}C with continuous shaking at 12000 rpm. 2 µL of digested sample was taken at 30, 60 and 120 min time intervals and analysed by LC-MS to check digestion. In each case the 2 µL of digested serum was further diluted 40 fold in 0.05% TFA with a final total volume of 80 µL (containing 6.25 pg IGNIS prime peptide-1 and 37.5 ng serum digest per µL of the digested sample). 1 µL of this diluted serum digest was analysed by LC-MS using the Top10 DDA method. The remaining digest at 120 min was subjected to reduction by adding 10 µL of 1 M dithiothreitol (DTT) for 30 min at 56 ºC with shaking. The reduced sample was allowed to cool before adding 10 µL of 1 M iodoacetamide (IAA) and incubating the sample in dark at room temperature for 1 hour. The digested, reduced and alkylated sample was diluted accordingly prior to LC-MS analysis.

Sample clean-up: Peptides were desalted using an online C18 trap column (PepMap 100, 5 µm particle size, 300 µm i.d. x 5 mm, Thermo Scientific). Desalting of 1 µL of digested sample was achieved using a loading buffer consisting of 0.05% v/v trifluroacetic acid (TFA) in water at a flow rate 12 µL/min for 4 min after which the mobile phase was diverted to the analytical column via a trap column to separate the peptides trapped on the trap column.

NAFLD samples: 4 control serum samples were collected from healthy volunteers at the John Radcliffe hospital, Oxford, UK. 11 serum samples from NAFLD patients were obtained from hospitals within the Imperial College Healthcare NHS Trust, London, UK (Supplementary Table 2). The ethics approval was given by the West London Research Ethics Committee; reference number 10/H0711/58.

Western blotting: Western blotting for 15 samples (4 controls and 11 NAFLD) was performed using an APO-F primary antibody (Goat IgG polyclonal; product number # SC-107408, Santa Cruz, USA).

Linearity of APO-F endogenous peptides: Using IGNIS the absolute concentration of endogenous peptides was calculated based on the concentration of the released custom heavy peptide which is equivalent to a one point calibration. Hence it was necessary to check the linearity of endogenous APO-F peptides in this calibration range. A digest of human serum was diluted using a digest of fetal calf serum (FCS) to keep the total matrix constant. Both the human and FCS digests were diluted to 200 ng/µL separately with water. Five different dilutions of human serum digest were prepared by diluting with FCS digest as follows (in each case the total protein concentration was 200 ng/µL and volume was 10 µL): (1) 8 µL of FCS digest + 2 µL of human serum digest (human protein concentration 40 ng/µL), (2) 6 µL of FCS digest + 4 µL of human serum digest (human protein concentration 80 ng/µL), (3) 4 µL of FCS digest + 6 µL of human serum digest (human protein concentration 120 ng/µL), (4) 2 µL of FCS digest + 8 µL of human serum digest (human protein concentration 160 ng/µL), (5) 10 µL of human serum digest (human protein concentration 200 ng/µL). 1 µL (200 ng total protein) of each sample was injected and analysed by LC-MS. The peak area of endogenous APO-F peptide-1 was acquired by scheduled PRM and was plotted against the total human serum protein concentration on column.

Supporting results:

SMART Digest™ digestion of a synthetic IGNIS peptide and human serum

In order to investigate the efficiency of the SMART Digest™ kit to digest IGNIS prime peptide-1 spiked into human serum, the released URP-1,heavy peptide-1 and undigested

IGNIS prime peptide-1 were targeted using PRM. Trypsin digested human serum (without spiked IGNIS prime peptide-1) was used as blank to confirm that the peaks of undigested IGNIS prime peptide-1, released heavy peptide-1 and URP-1 (iB) were free from interferences (Supplementary Figure S4 and S5). Undigested IGNIS prime peptide-1 was spiked into the same blank sample (6.25 pg IGNIS prime peptide-1/37.5 ng serum digest) to check presence of IGNIS prime peptide-1 (Supplementary Figure S4). IGNIS prime peptide-1 was spiked into human serum (6.25 pg IGNIS prime peptide-1/37.5 ng human serum) and was digested with the SMART Digest™ kit according to the manufacturer's protocol. Digested samples were collected at the time intervals of 30, 60 and 120 min to check digestion of human serum and IGNIS prime peptide-1. Digestion of IGNIS prime peptide-1 was assessed by analysing the peak areas of released heavy peptide-1 and URP-1 (iB) from IGNIS prime peptide-1 (Supplementary Figure S5) and the absence of IGNIS prime peptide-1 peak (Supplementary Figure S4) at time intervals 30, 60 and 120 min. There was no marked difference in the peak areas of released URP-1 and heavy peptide-1 after digesting for 30, 60 and 120 min suggesting that the IGNIS prime-1 was completely digested at or before 30 min. Digestion of human serum (300 µg total protein) using the SMART Digest™ kit was evaluated by measuring the number of observed charges (z = +2, +3, +4, +5, +6) after digesting for 30, 60, 120 min and at 120 min with reduction-alkylation using the Top10 DDA method. There was no marked difference in the number of observed charges for each of these five charges among the different digestion times. However when reducing and alkylating after digesting for 120 min the number of observed +2, +3 charges were higher and the number of observed +4, +5 and +6 charges were lower in comparison to 30, 60 and 120 min without reduction and alkylation (Supplementary Figure S6). The increased number of +2 and +3 charges and decreased number of +4, +5 and +6 charges are due to cleavage of the di-sulphide bonds between two joined peptides after reduction and alkylation since separated peptides have a smaller size and therefore carry less charge.

The SMART Digest™ trypsin kit provides complete digestion of synthetic IGNIS prime peptide-1 spiked into human serum and is considerably faster than in-solution digestion where IGNIS prime peptides and human serum were digested separately (as discussed in the Results and discussion section).

Limit of detection (LOD) for conventional absolute quantitation

The limit of detection for the conventional way of absolute quantitation was obtained by spiking a serial dilution of synthetic heavy peptide-1 (0.01, 0.05, 0.1, 0.2 and 0.5 fmol/µL) into a digest of serum. The lowest detectable concentration was 0.05 fmol/µL with an acceptable dotp value (≥0.7) and mass error ≤ 5 ppm (See supplementary Figure S10).

Reference

1. Kumar Abhinav GB, Zitzmann Nicole. Multiple reaction monitoring and multiple reaction monitoring cubed based assays for the quantitation of apolipoprotein f. Journal of chromatography B 2016;1033-1034:278-86.

Supplementary Figures

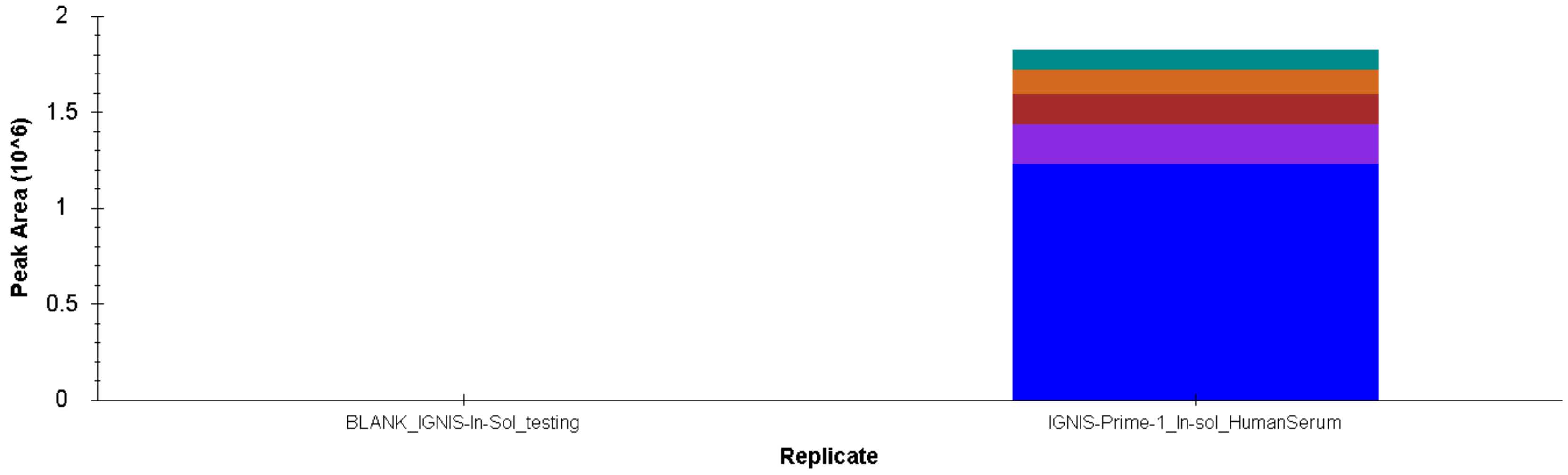

Supplementary Fig. S1. Undigested IGNIS prime-1 detected after following the optimised in-solution trypsin digestion of human serum spiked with IGNIS prime-1.

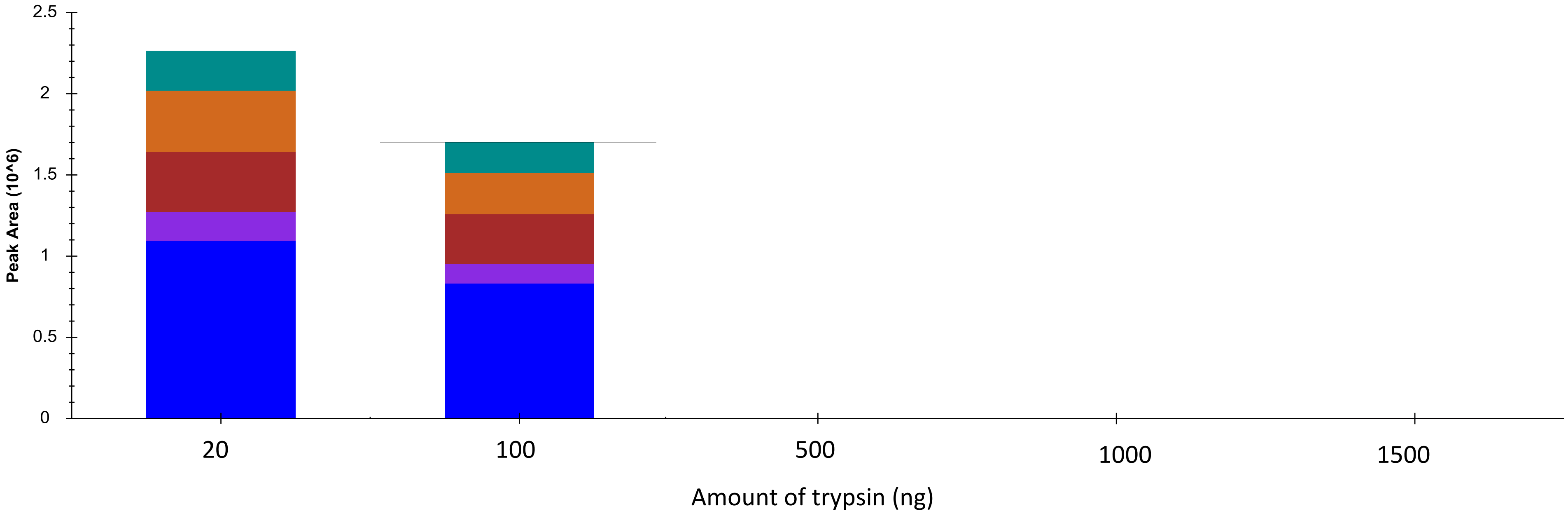

Supplementary Fig. S2. The presence of undigested IGNIS prime 1 at lower trypsin concentrations (20 and 100 ng). The presence of full length IGNIS prime-1 (SLPTEDC[+57]ENEKAALPAAFK, underlined amino acids are heavy labelled C^{13}/N^{15}) is high at 20 ng and it decreases with increasing amounts of trypsin. The presence of undigested IGNIS prime disappears at 500-1500 ng trypsin.

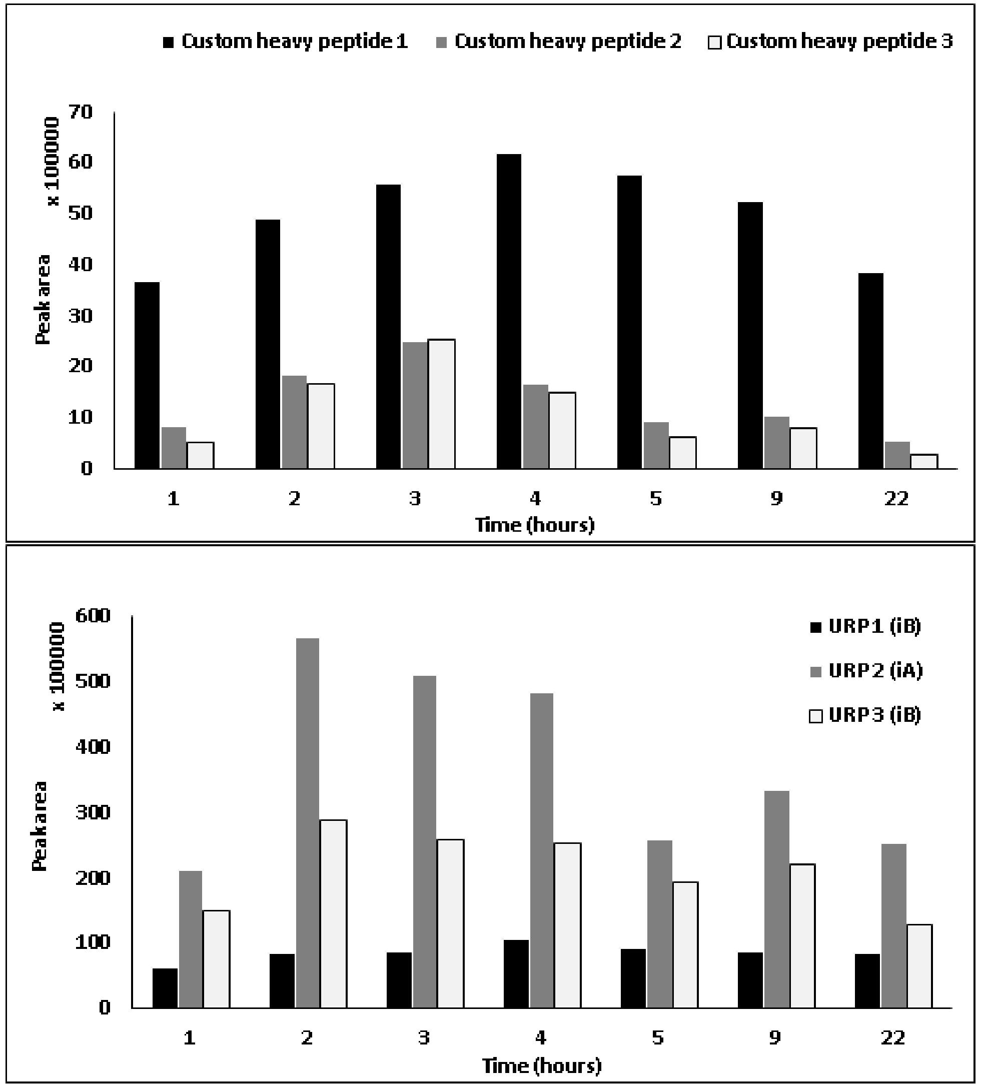

Supplementary Fig. S3. The optimum time required for the complete digestion of IGNIS prime peptides 1, 2 and 3. The upper and lower panels show the peak area of the released custom heavy peptides and URPs from IGNIS prime peptides, respectively, over the 22 hours of trypsin digestion. IGNIS prime 1 was digested with 500 ng of trypsin and the optimum time required for digestion was 4 hours. IGNIS prime peptides 2 and 3 were digested with 1 µg of trypsin and the optimum time required for complete digestion was between 2-3 hours.

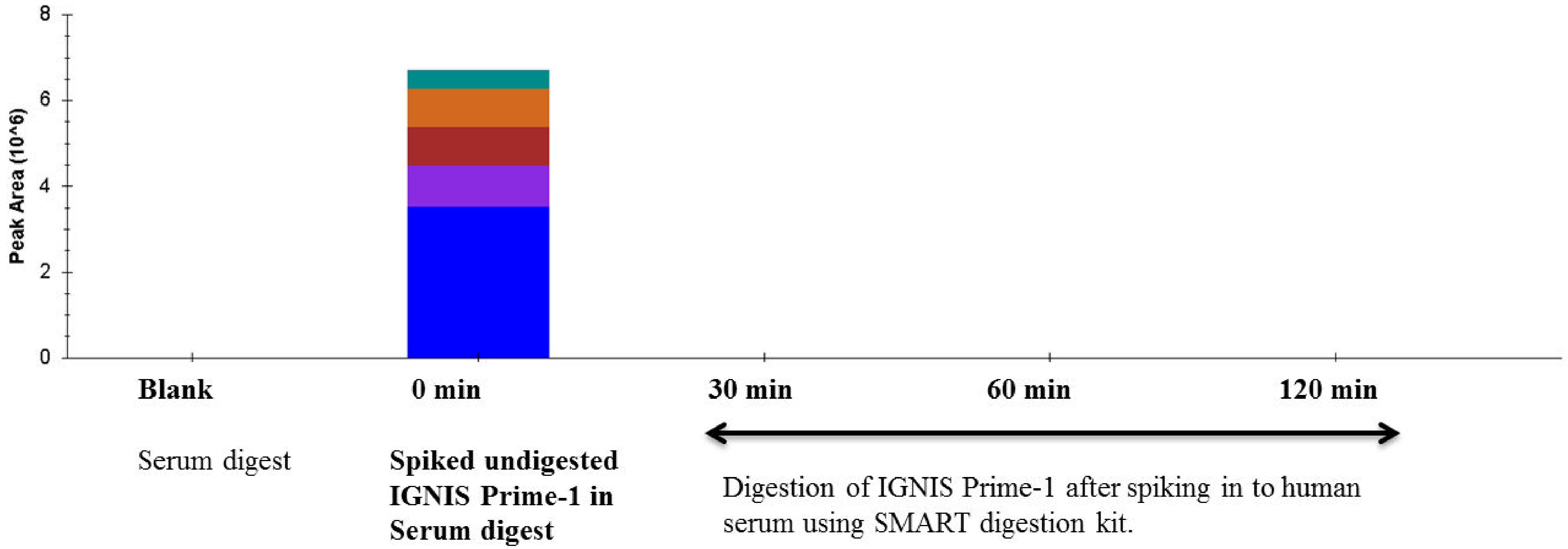

Supplementary Fig. S4. The detection of IGNIS prime-1 (6.25 pg/37.5 ng human serum proteins; 24 ng IGNIS prime-1/300 ng human serum) at 0 min and the absence of IGNIS prime-1 in blank and samples digested for 30, 60 and 120 min using the SMART Digest™ kit. Complete digestion of IGNIS prime-1 in the presence serum proteins was achieved in just 30 min which is considerably quicker than in-solution digestion. During in-solution trypsin digestion IGNIS prime peptide-1 was not digested in the presence of human serum proteins and when IGNIS prime-1 was digested separately it required 500 ng of trypsin and 2-3 hours of digestion time (Supplementary Figure S1 and S2 respectively).

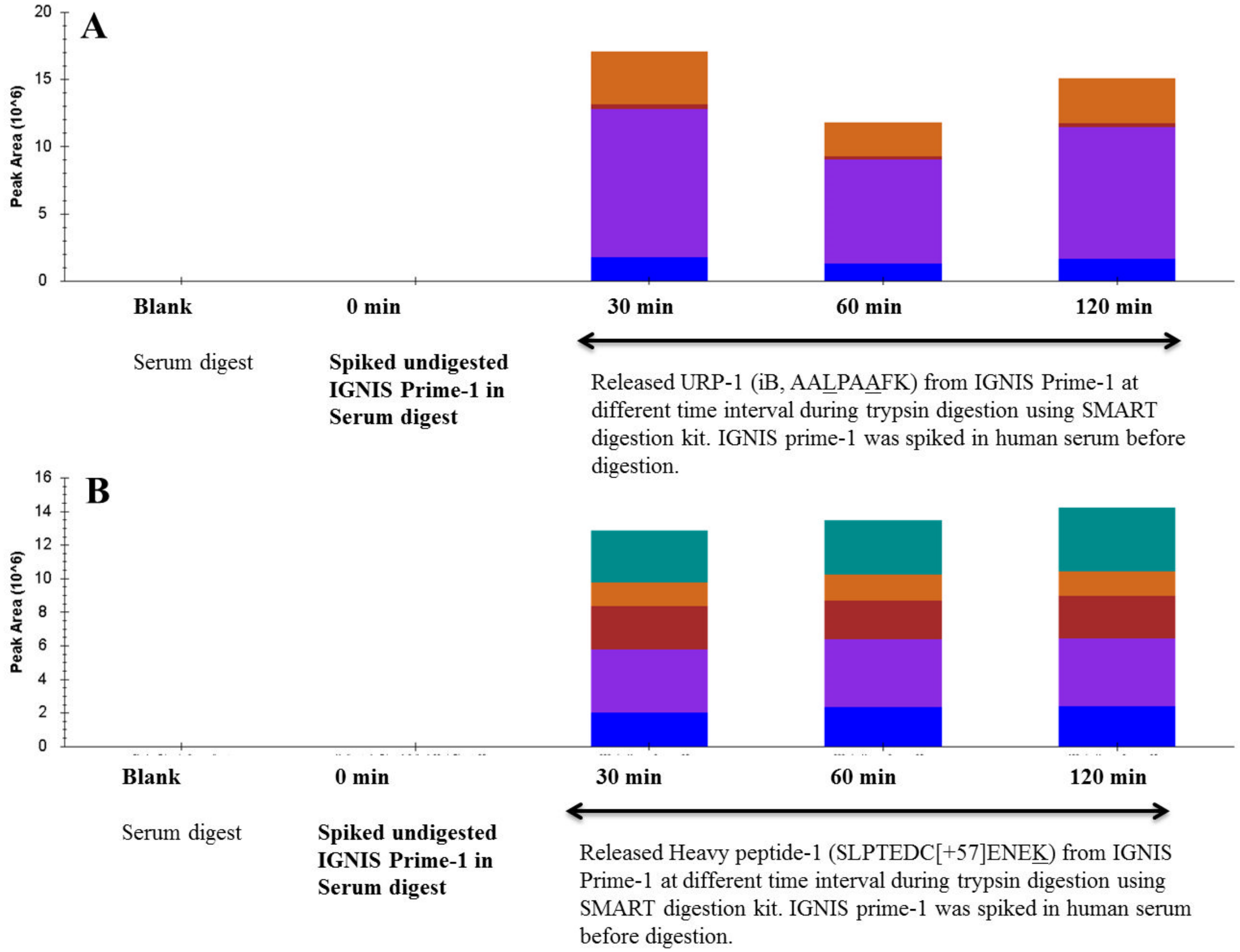

Supplementary Fig. S5. The detection of released URP-1(A) and heavy peptide-1 (B) from digested IGNIS prime-1 (6.5 pg/37.5 ng human serum proteins; 24 ng IGNIS prime-1/300 µg total human serum) in samples digested for 30, 60 and 120 min using the SMART Digest™ kit. The peak areas of released URP-1 and heavy peptide-1 are fairly constant at 30, 60 and 120 min digestion time which shows that the IGNIS prime peptide-1 was completely digested at/before 30 min. As described in Supplementary Fig. S4, the SMART Digest™ kit provides complete and faster digestion of IGNIS prime peptide-1 in the presence of complex human serum matrix in comparison to in-solution digestion.

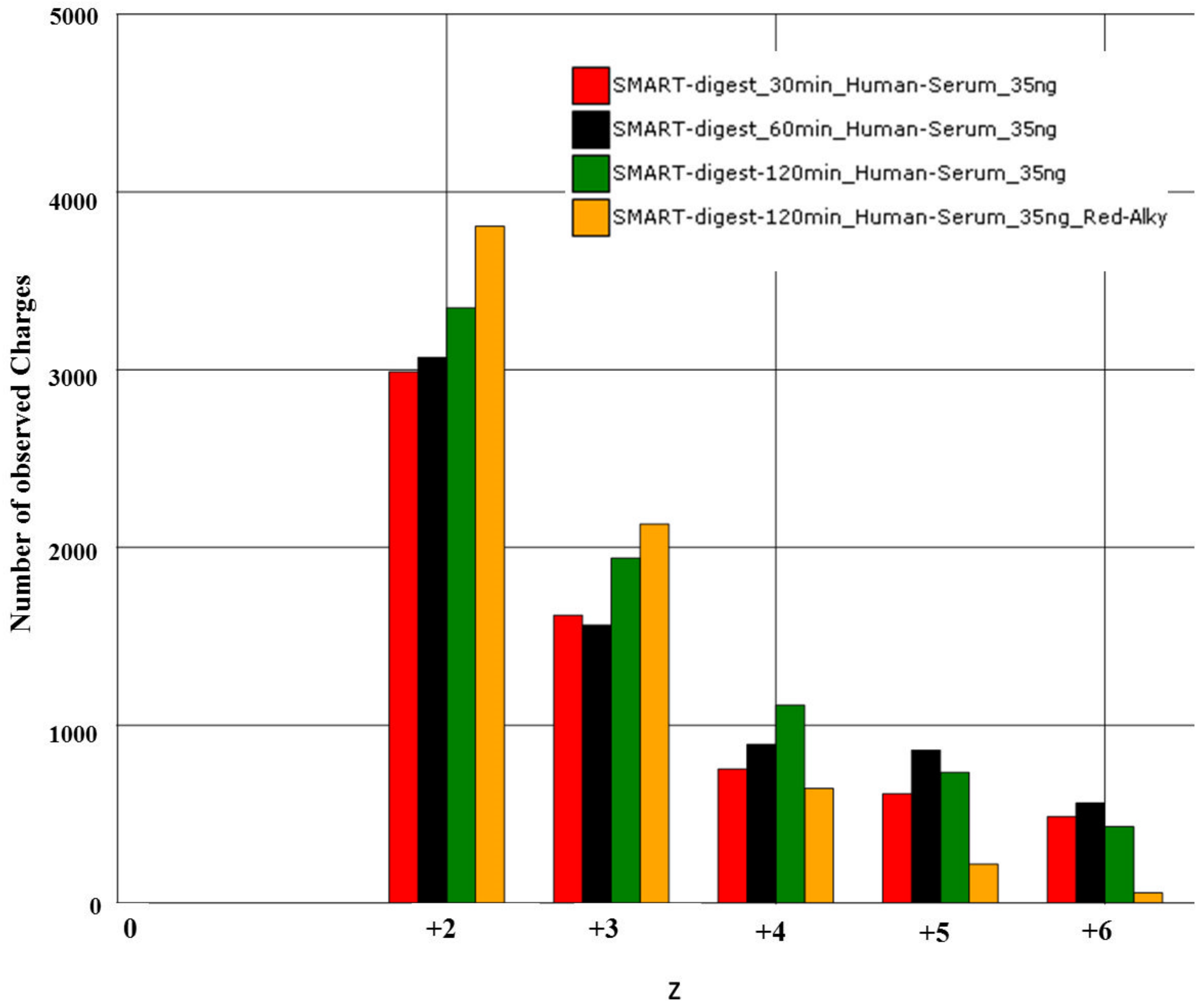

Supplementary Fig. S6. The number of observed charges (+2, +3, +4, +5 and +6) after digesting for 30, 60 and 120 min using the SMART Digest™ kit. There is no marked difference in the number of observed charges for each of these five charges among the different digestion times except when using reduction and alkylation. This constant number of charges at 30, 60 and 120 min (without reduction and alkylation) shows that digestion was completed at 30 min. The number of +2 and +3 charges increased and the number of +4, +5 and +6 charges decreased after reduction and alkylation which is due to cleaved disulphide bonds between cysteine containing peptides resulting into smaller size peptide carrying smaller charges.

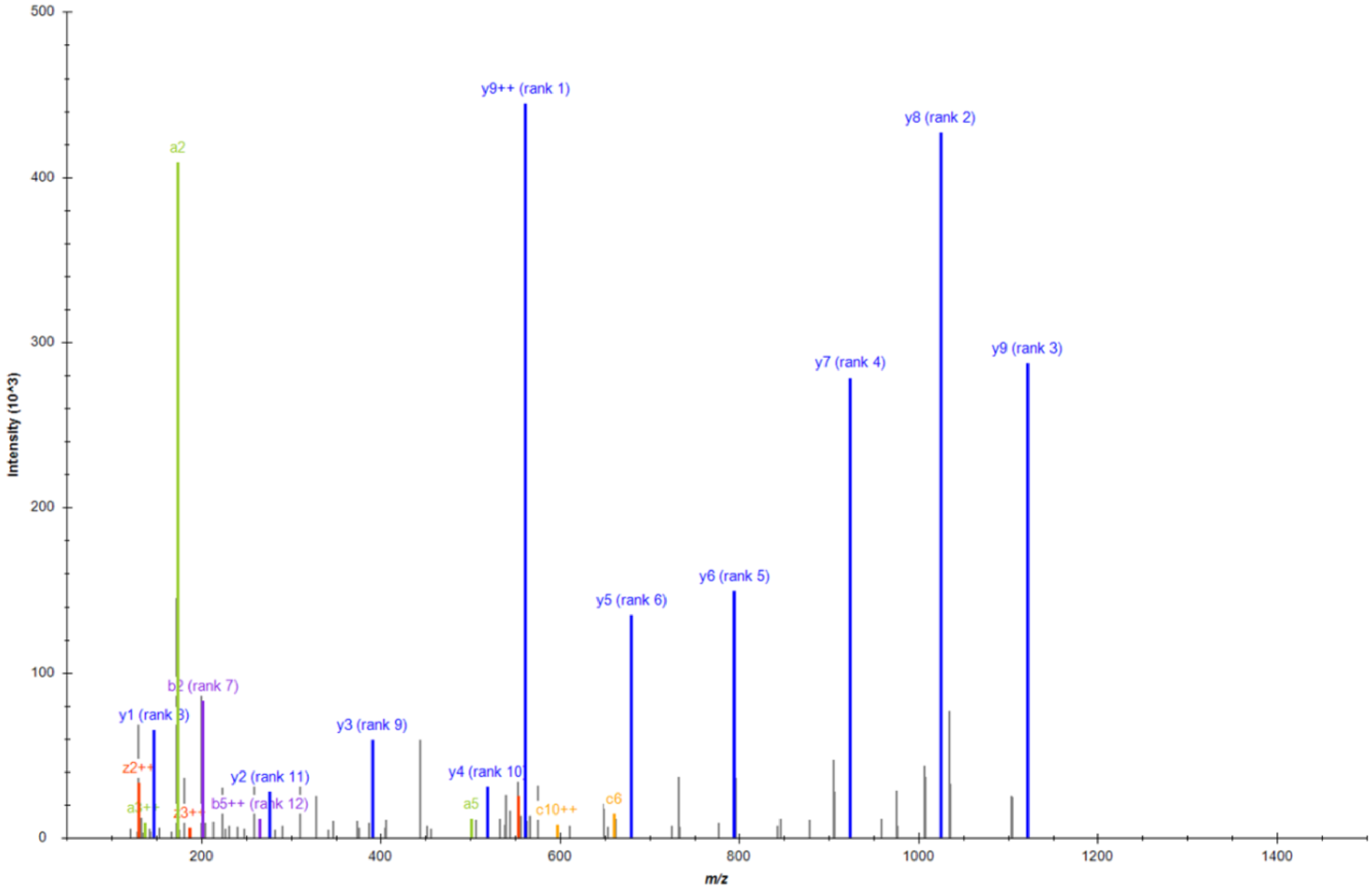

Supplementary Fig. S7. Reference library of peptide SLPTEDC[+57]ENEK (peptide 1, z = +2). A light synthetic peptide for peptide 1 was spiked into a digest of a control human serum sample and data were acquired in data dependent acquisition mode on a Q Exactive mass spectrometer.

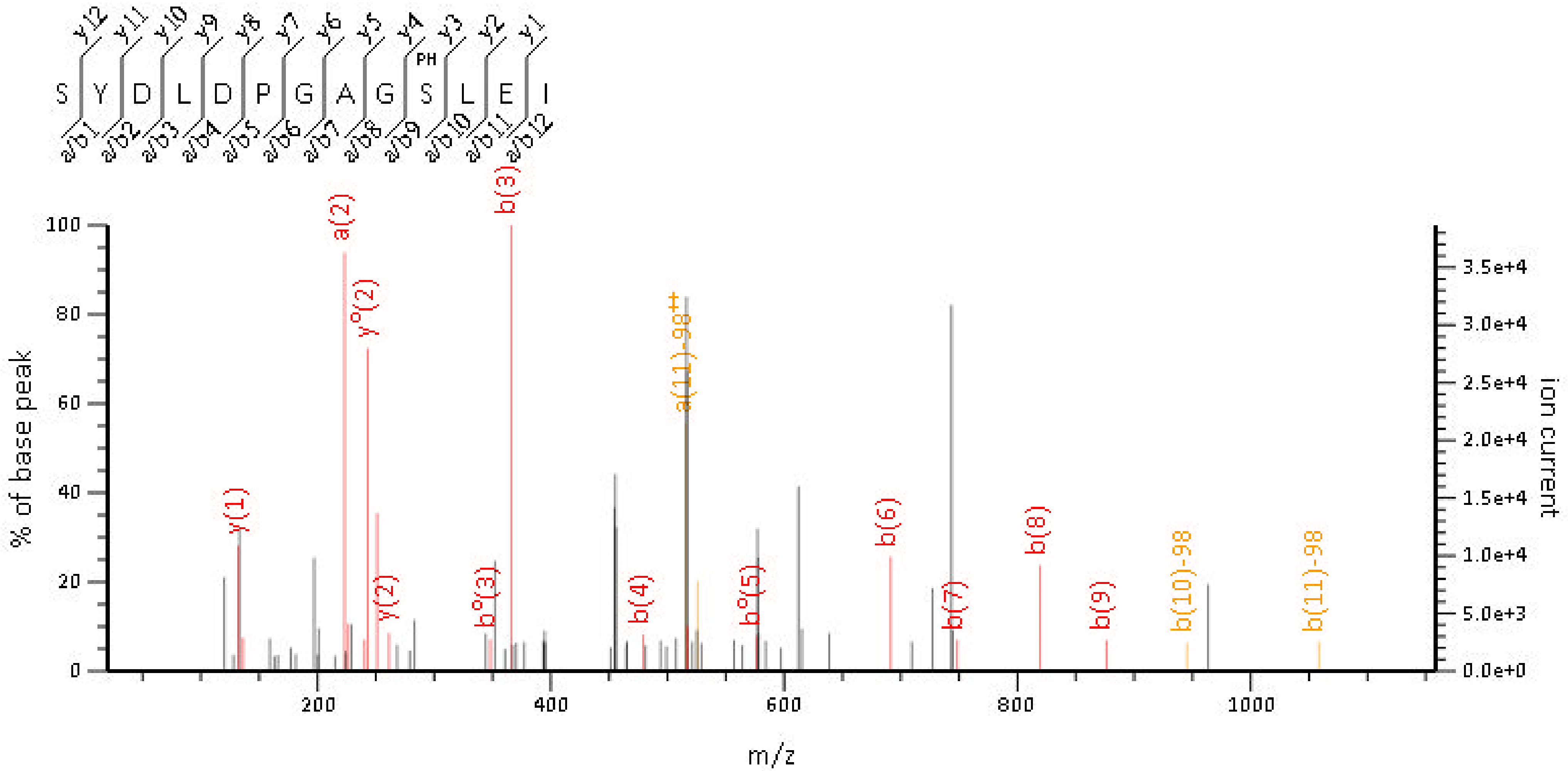

Supplementary Fig. S8. Detection of APO-F peptide 3 (314SYDLDPGAGSLEI326) and presence of phosphorylation of serine at position 323. A 1st dimension HILIC fraction at pH 6.5 was collected from a digest of human plasma and the same fraction was analysed on a reversed phase C18 nano-flow LC column in 2nd dimension. The separated peptides from the 2nd dimension were acquired on Q Exactive mass spectrometer using Top10 data dependent acquisition (DDA). The peptide search was performed on the Mascot search engine using the Uniprot database and phosphorylation of serine/threonine was set as a variable modification.

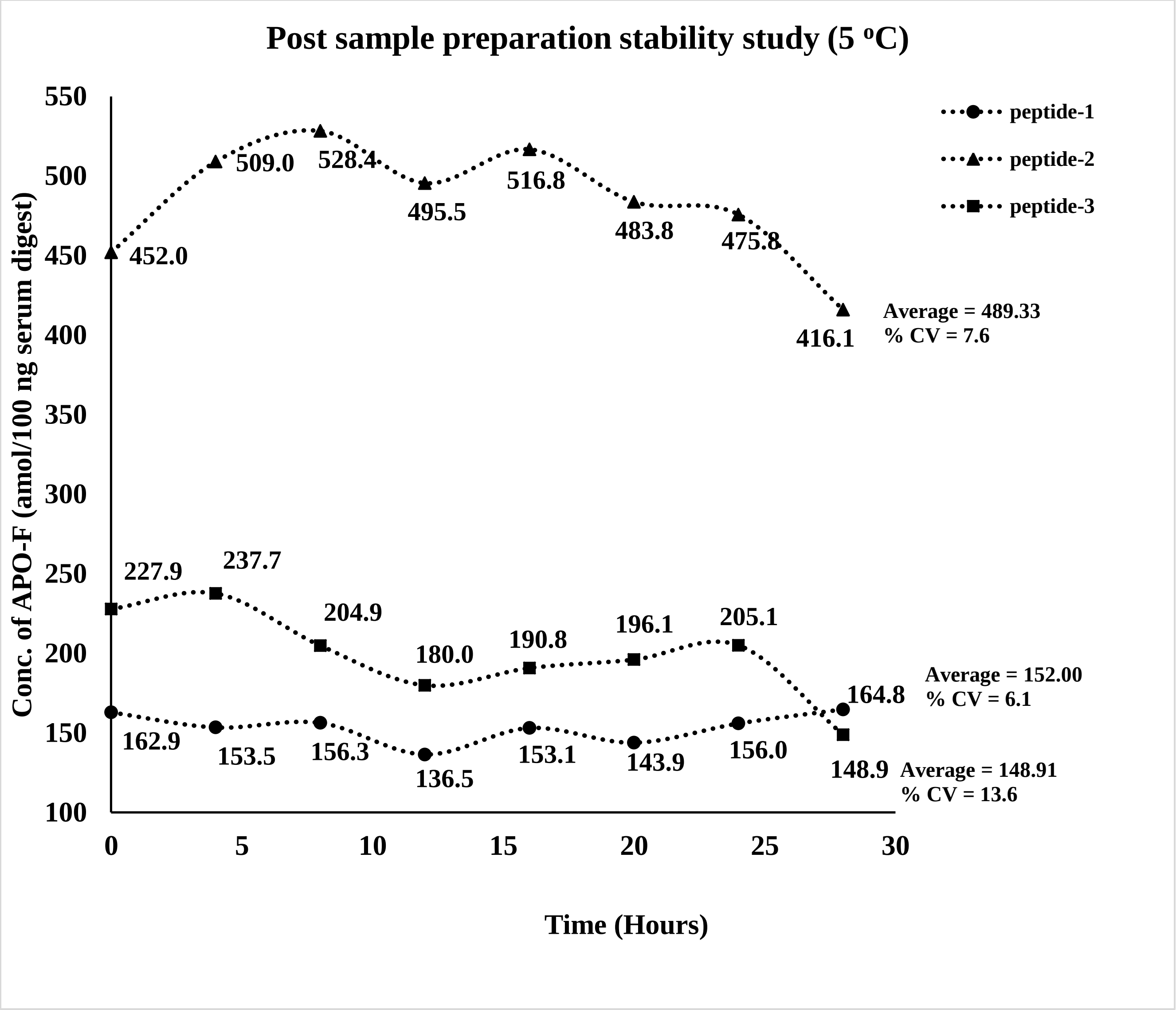

Supplementary Fig. S9. The post sample preparation stability at 5 ^{o}C over a 28 hour time period. Peptides 2 and 3 showed decrease in their concentration at 28 hours, however, within 24 hours the concentrations were fairly constant. This suggests that the IGNIS method can be used for high throughput analysis within 24 hours of sample preparation.

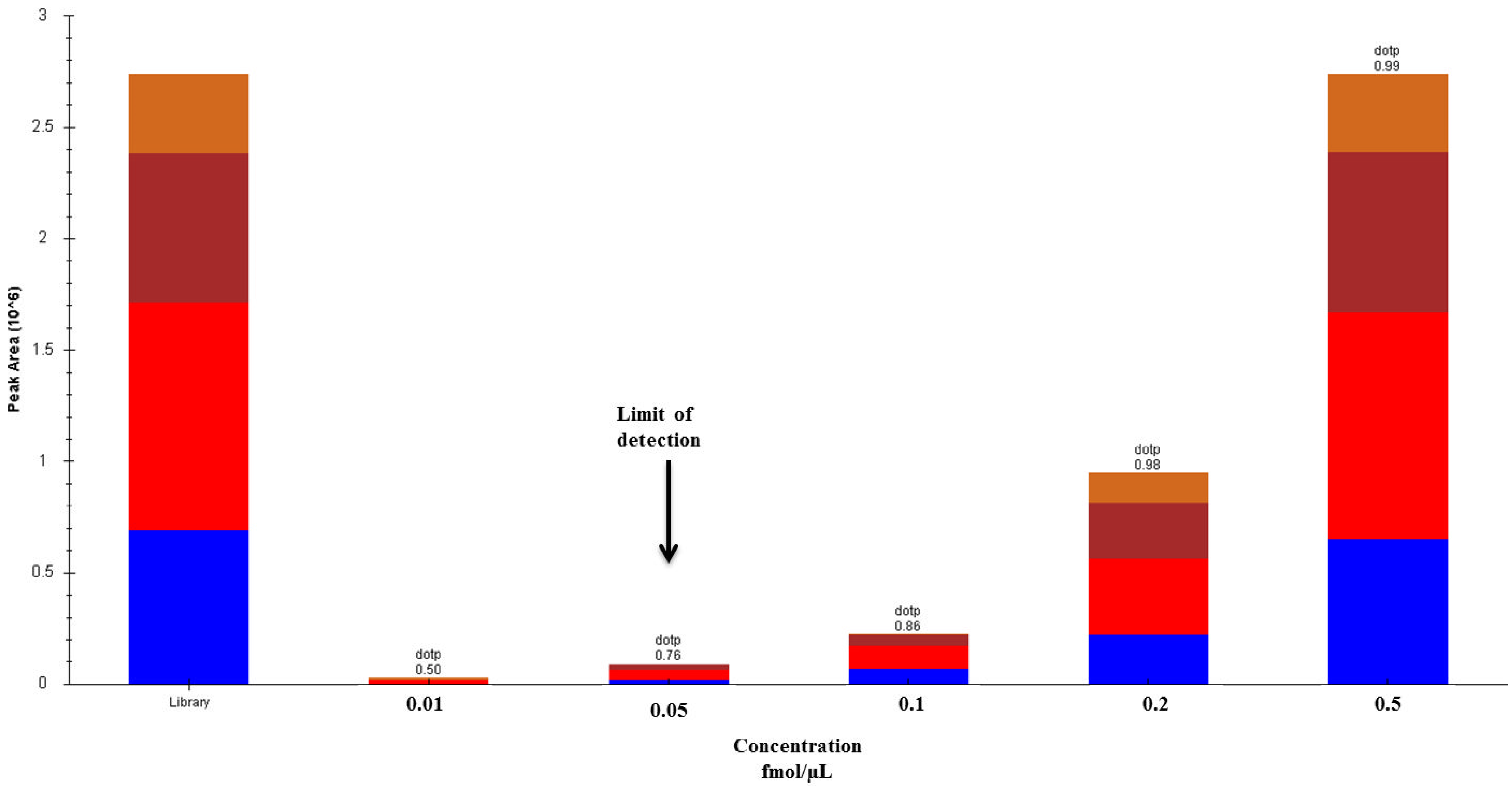

Supplementary Figure S10: Showing limit of detection (LOD) of peptide-1 (APO-F) after spiking the custom heavy peptide-1 into the digest of serum (100ng/uL). The LOD was found to be 0.05 fmol/uL as 0.01 fmol/uL concentration has very low dotp value which indicates the signal is due to background noise.

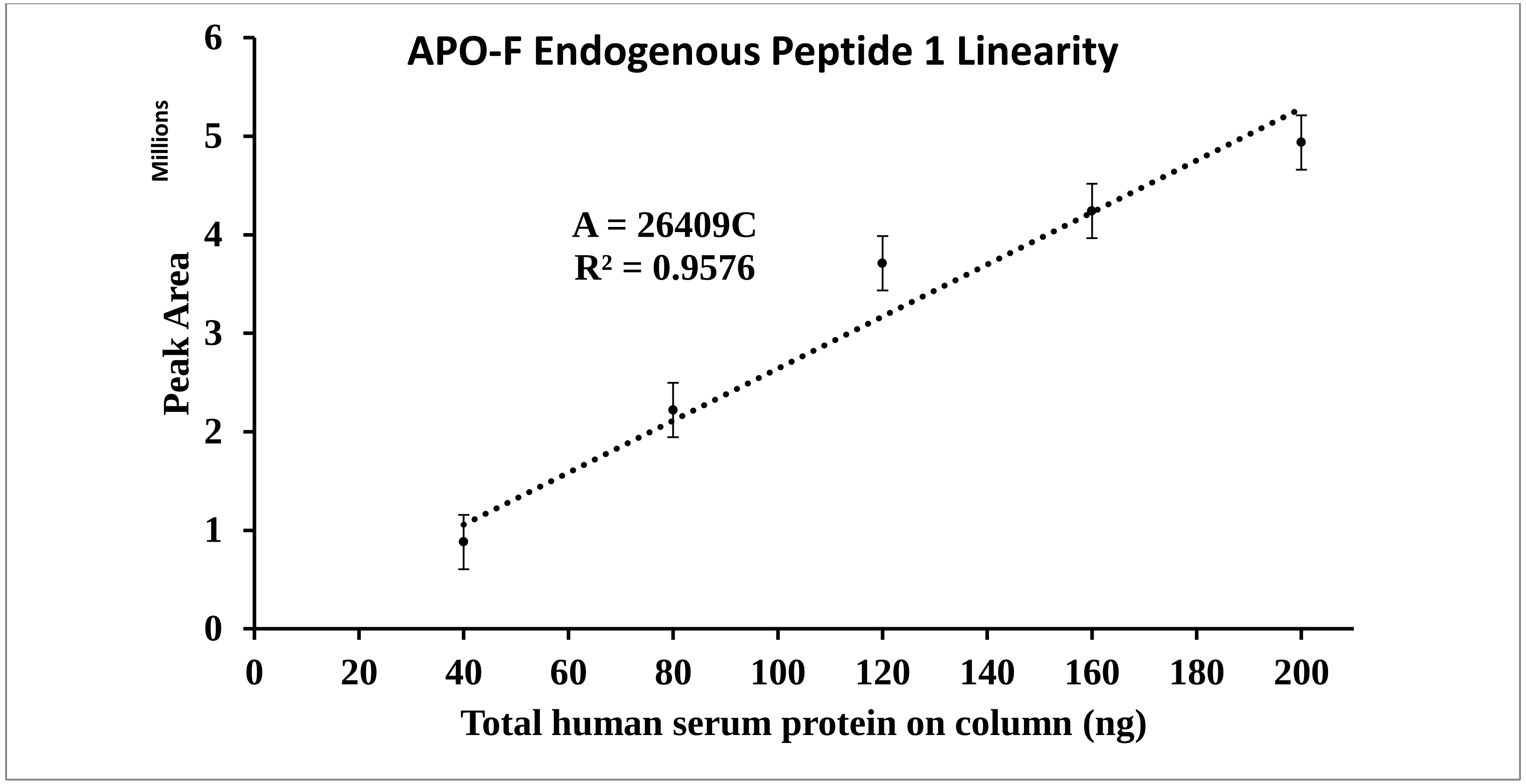

Supplementary Figure S11: Linearity of endogenous peptide-1 when 40-200 ng/µL of human serum digest was injected and analysed by LC-MS. The IGNIS based method shows linearity for the IGNIS isotopologues and the calculated concentration of the endogenous peptide is equivalent to a one point calibration. Linearity for the endogenous peptide shows that its concentration can be calculated using IGNIS as long as its concentration falls within this linearity range of endogenous peptide. 200 ng/µL of digested human serum was diluted with 200 ng/µL of blank matrix (fetal calf serum digest) to achieve 40-200 ng/µL of human serum (keeping the total matrix constant at 200 ng/µL). 1 µL of each diluted sample was injected which gave 40-200 ng on column of human serum digest. See the Supplementary Methods section for more details on sample preparation. A = Peak area; C = serum protein (ng).

Supplementary Tables

Supplementary Table 1. Concentration of iDCM-8 isotopologues after reconstitution in 300 µL reconstitution solvent. Isotopically heavy labelled amino acids are underlined.

Dilution table for iDCM-8		Concentration after reconstitution in a volume of 300 µL	Amount on column after 2.73 fold dilution and 1 µL LC-MS injection
Name	Isotopologues	amol/µL	amol
iC	AALPAAFK	109350.00	40054.95
iD	AALPAAFK	36450.00	13351.65
iE	AALPAAFK	12150.00	4450.55
iF	AALPAAFK	4050.00	1483.52
iG	AALPAAFK	1350.00	494.51
iH	AALPAAFK	450.00	164.84
iJ	AALPAAFK	150.00	54.95
iK	AALPAAFK	50.00	18.32

Supplementary Table 2: NAFLD serum sample information.

Sample number	Sample	Date sample taken	Age at time of sampling	Sex	Fibrosis stage (Kleiner/Brunt x/4)	Diabetic	CVD	NASH vs no NASH	Ethnicity	Alanine aminotransferase (IU/L)	Albumin (g/L)	Alkaline phosphatase (IU/L)	Bilirubin (mmol/L)	Creatinine (mmol/L)	Gamma glutamyl transpeptidase (IU/L)	HCV genotype	Platelet count	Date of liver biopsy	Number of tracts	Size of the core (mm)	NAS score (out of 8)	Ballooned hepatocytes? (1/0)
10	NAFL	19/09/2012	50	M	0	N	0	0	Indian	76	39	86	30	72	37	N/A	277	06/03/2012		40	2	0
11	NAFL	13/09/2012	43	M	0	N	0	0	White non-British	114	54	68	33	NK	71	N/A	162	11/04/2012		20		0
12	NAFL	13/09/2012	51	M	0	N	0	0	White non-British (Italian)	31	42	60	12	77	72	N/A	194	20/03/2012	14	24	3	0
4	NASH F0	16/04/2012	59	M	0	N	0	1	White Brit	61	43	101	8	74	488	N/A	249	06/10/2011		35		1
9	NASH F0	30/08/2011	33	M	0	N	0	1	South East Asian (Filipino)	110	41	90	11	79	96	N/A	317	23/09/2010				1
3	NASH F1	14/09/2011	53	M	1	Y	0	1	White Non-British (Portuguese)	54	38	75	12	68	104	N/A	209	22/02/2011		20		1
8	NASH F1	23/07/2012	52	M	1	Y	0	1	Middle Eastern (Iranian)	39	44	60	34	89	23	N/A	175	08/03/2012		30	4	1
2	NASH F3	05/09/2011	40	M	3	N	0	1	Middle Eastern	89	39	69	11	76	67	N/A	221	21/09/2010		17		1
5	NASH F3	18/06/2012	58	M	3	Y	0	1	East Asian (Chinese)	59	44	66	14	70	81	N/A	191	13/12/2011		45		1
6	NASH F3	31/10/2011	46	M	3	Y	0	1	South Asian (Sri Lankan)	118	46	72	9	87	113	N/A	221	16/06/2011		25		1
7	NASH F3	13/06/2011	32	M	3	N	0	1	White Non-British (Italian)	128	47	102	12	82	136	N/A	289	22/03/2011		20		1

Supplementary Table 3. The peak area ratios of spiked light peptide 1 and heavy peptide 1 in a digest of fetal calf serum which were used to obtain a six point calibration curve for the conventional way of quantification. Light peptide 1 was spiked in different concentrations (ranging from 0.2-7.5 fmol/µL) and heavy peptide 1 was spiked at a fixed concentration (0.4 fmol/ µL) into 100 ng/µL of digested fetal calf serum. The %CV of precision for three repeat injections were in the range 0.73-10.9%. The accuracy of the quality control samples at the lower (0.3 fmol/µL) and middle (3.75 fmol/µL) regions of the calibration curve were 86.3% and 87.6%, respectively. The absolute amount of APO-F in digest of human serum was found to be 147.7 fmol/100 ng of human serum digest. % accuracy shows how close the calculated concentration of light AQUA peptides are to their actual known concentration.

Samples per µL	Peak area of light peptide			Peak area of heavy peptide			Peak area ratio (Light/Heavy)					
	Replicate-1	Replicate-2	Replicate-3	Replicate-1	Replicate-2	Replicate-3	Replicate-1	Replicate-2	Replicate-3	Average	Standard deviation	%CV
Cal-1 0.2 fmol	944674.27	1050074.78	967774.91	2216844.63	2809002.19	2395982.75	0.43	0.37	0.40	0.40	0.03	6.54
Cal-2 0.5 fmol	3372867.59	3035575.44	3200921.69	2229305.72	2487073.00	2230731.34	1.51	1.22	1.43	1.39	0.15	10.90
Cal-3 1.5 fmol	11178834.50	11981624.00	11771119.63	2138539.06	2468921.34	2158030.91	5.23	4.85	5.45	5.18	0.30	5.87
Cal-4 2.5 fmol	19794151.00	22450080.00	21761720.75	2226536.34	2504842.66	2321868.75	8.89	8.96	9.37	9.08	0.26	2.87
Cal-5 6.25 fmol	57013819.00	63729327.00	62238164.00	2403663.66	2656874.34	2630679.47	23.72	23.99	23.66	23.79	0.17	0.73
Cal-6 7.5 fmol	66671099.00	74767242.00	72194184.00	2218402.28	2383893.91	2362320.53	30.05	31.36	30.56	30.66	0.66	2.15
QC 0.3 fmol	2652506.70	2451311.03	2693281.03	2416126.47	2504797.13	2541353.63	1.10	0.98	1.06	1.05	0.06	5.82
QC 3.75 fmol	32009722.50	32149120.50	34956532.00	2550734.13	2431144.31	2678916.13	12.55	13.22	13.05	12.94	0.35	2.71
Human serum 100 ng/µL	1393768.92	1173690.66	1201110.05	2434056.34	1933419.72	2110697.22	0.57	0.61	0.57	0.58	0.02	3.60

Equation of line: A = 3.938C, R^2 = 0.9954 | A = ratio of peak area (Light/Heavy) and C = concentration of light peptide on column (fmol/µL).

Absolute value of light peptide in QC samples.

	Calculated conc. (fmol/µL)	% Accuracy	
QC (0.3 fmol/µL)	0.26	86.66	Absolute value of APO-F (using peptide 1) in 100 ng of normal serum digest (100 ng/µL) on column = 0.1477 fmol (147.7 amol).
QC (3.75 fmol/µL)	3.29	87.73	

Supplementary Table 4: The peak area of the iDCM-8 isotopologues, released peptides from IGNIS prime 1 and endogenous light peptide 1 in the spiked digest of human serum. The peak area of only six iDCM-8 isotopologues were used to plot a calibration curve. The same sample was injected three times to find the %CV for precision. The measured peak area of the released URP 1 and heavy peptide 1 from IGNIS prime 1 were used to calculate the absolute concentration of APO-F (endogenous peptide 1) in a digest of normal human serum (100 ng on column).

Peptides	Peak area of peptide in spiked human serum (100 ng on column)					
	Replicate-1	Replicate-2	Replicate-3	Average	Standard deviation	%CV
AALPAAFK (iC)	122600074.00	128520276.00	118888428.00	123336259.33	4857942.01	3.94
AALP**AA**F**K** (iD)	46131490.50	53469949.50	49976540.00	49859326.67	3670633.37	7.36
AA**L**PA**AF**K (iE)	19092008.00	17674887.88	16404057.50	17723651.13	1344638.56	7.59
AALP**AAFK** (iF)	5824416.19	5359992.50	4987008.81	5390472.50	419534.92	7.78
AA**LPAAF**K (iG)	1518323.34	1503972.69	1655396.25	1559230.76	83590.29	5.36
A**A**L**PAAFK** (iH)	631358.51	647926.28	522088.81	600457.87	68373.27	11.39
AA**L**PA**A**FK (iB)<br>Released UPR 1	910483.55	901176.21	846441.83	886033.86	34602.07	3.91
SLPTEDC[+57]ENE**K**<br>Released heavy peptide 1	1498175.20	1274228.28	1432361.61	1401588.36	115101.26	8.21
SLPTEDC[+57]ENEK<br>(Endogenous light peptide 1)	758478.88	648519.80	737869.70	714956.13	58451.04	8.18
**Calculation of concentrations (amol/μL) of URP, heavy peptide and endogenous light peptide 1**						
	Replicate-1	Replicate-2	Replicate-3	Average	Standard deviation	%CV
Concentration of URP	292.36	273.41	277.24	281.00	10.02	3.57
Concentration of heavy peptide 1	292.36	273.41	277.24	281.00	10.02	3.57
Concentration of endogenous light peptide 1	148.01	139.15	142.82	143.33	4.45	3.11

Formulae to calculate the concentration of endogenous peptides:

$$\text{Conc. of URP 1} = \frac{\text{Peak area of URP 1}}{\text{Equation of line}}$$

Equation of line: Replicate-1:A = 3114.3C, Replicte-2: A = 3296.1C and Replicate-3: A = 3053.1C

$$\text{Conc. of Heavy peptide 1} = \text{Conc. of URP 1}$$

$$\text{Conc. of endogenous light peptide 1 (i. e conc. of APO-F)} = \left(\frac{\text{Conc. of heavy peptide 1}}{\text{Peak area of heavy peptide 1}}\right) \text{peak area of endogenous light peptide 1}$$

Supplementary Table 5. Summary of the technical repeats, inter sample and inter day precisions of the absolute concentration of APO-F using peptides 1, 2 and 3 by the IGNIS method of absolute quantitation.

Technical repeat precision (n = 3)									
Absolute concentration of APO-F (amol/100 ng serum digest)									
	Replicate 1	Replicate 2	Replicate 3	Average	STDEV	%CV			
Peptide 1	148.01	139.15	142.82	143.33	4.45	3.11			
Peptide 2	423.22	482.77	371.63	425.87	55.62	13.06			
Peptide 3	327.39	337.99	253.61	306.33	45.96	15.00			
Inter-day variation (sample preparation on three different days from the same stock of serum)									
Absolute concentration of APO-F (amol/100 ng serum digest)									
	Day 1	Day 2	Day 3						
	December 2014	March 2015	April 2015	Average	STDEV	%CV			
Peptide 1	187.41	143.28	161.07	163.92	22.20	13.55			
Peptide 2	419.93	508.81	425.87	451.54	49.69	11.00			
Peptide 3	212.12	187.26	306.33	235.24	62.81	26.70			
Inter-sample variation (sample preparation from six different sample stocks on three different days)									
Absolute concentration of APO-F (amol/100 ng serum digest)									
	Sample 1	Sample 2	Sample 3	Sample 4	Sample 5	Sample 6	Average	STDEV	%CV
	December 2014	December 2014	December 2014	December 2014	March 2015	April 2015			
Peptide 1	217.87	169.18	175.18		143.28	161.07	173.32	27.65	15.95
Peptide 2	357.28	455.92	388.01	478.51	508.81	425.87	435.73	56.76	13.03
Peptide 3	181.75	205.86	169.45	291.42	187.26	306.33	223.68	59.60	26.65

Supplementary Table 6: Statistics based on the absolute concentration of three APO-F peptides to differentiate the stages of NAFLD.

Statistics of peptide-1 absolute concentration in NAFLD samples.											
	absolute concentration			absolute concentration			absolute concentration			absolute concentration	
Sample	Control	NAFL		NAFL	NASH F0		NASH F0	NASH F1		NASH F1	NASH F3
1	217.87	114.20		114.20	113.22		113.22	79.32		79.32	49.33
2	169.18	164.42		164.42	93.84		93.84	87.54		87.54	65.75
3	175.18	141.45		141.45							70.12
4											69.63
	t-test	0.088157		t-test	0.166793986		t-test	0.196317		t-test	0.063796
Sample	NAFL	NASH		Control/NAFL	NASH		NASH samples = 4 NASH F3 + 2 NASH F1 + 2 NASH F0 (total 8 samples)				
1	114.20	113.22		217.87	113.22						
2	164.42	93.84		169.18	93.84						
3	141.45	79.32		175.18	79.32						
4		87.54		114.20	87.54						
5		49.33		164.42	49.33						
6		65.75		141.45	65.75						
7		70.12			70.12						
8		69.63			69.63						
	t-test	0.001901		t-test	0.000079						

.

Statistics of peptide-2 absolute concentration in NAFLD samples.								
	absolute concentration			absolute concentration			absolute concentration	
Sample	Control	NAFL		NAFL	NASH F0		NASH F0	NASH F1
1	357.28	286.03		286.03	230.85		230.85	197.47
2	455.92	426.5		426.5	149.24		149.24	162.67
3	388.01	244.57		244.57				
4	478.51							
	t-test	0.136957		t-test	0.193790856		t-test	0.842969
Sample	NAFL	NASH		Control/NAFL	NASH		NASH F1	NASH F3
1	286.03	230.85		357.28	230.85		197.47	139.87
2	426.5	149.24		455.92	149.24		162.67	133.29
3	244.57	197.47		388.01	197.47			126.83
4		162.67		478.51	162.67			134.41
5		139.87		286.03	139.87		t-test	0.015082
6		133.29		426.5	133.29			
7		126.83		244.57	126.83			
8		134.41			134.41			
	t-test	0.002112		t-test	0.000021			

Sample	NASH F0	NASH F3	
1	230.85	139.87	
2	149.24	133.29	
3		126.83	
4		134.41	
	t-test	0.08958	

NASH samples = 4 NASH F3 + 2 NASH F1 + 2 NASH F0 (total 8 samples)

Statistics of peptide-3 absolute concentration in NAFLD samples.

	absolute concentration			absolute concentration			absolute concentration	
Sample	Control	NAFL		NAFL	NASH F0		NASH F0	NASH F1
1	181.7534	114.0725		114.0725146	102.188		102.188	67.32811
2	205.8647	152.5673		152.5673475	53.17256		53.17256	63.38313
3	169.4514	149.6953		149.6953309				
4	291.4191							
	t-test	0.084419		t-test	0.086391		t-test	0.665916

	absolute concentration			absolute concentration			absolute concentration	
Sample	NAFL	NASH		Control/NAFL	NASH		NASH F1	NASH F3
1	114.0725	102.188		181.753397	102.188		67.32811	79.69309
2	152.5673	53.17256		205.8646887	53.17256		63.38313	68.22713
3	149.6953	67.32811		169.4514466	67.32811			73.07209
4		63.38313		291.4190989	63.38313			77.7064
5		79.69309		114.0725146	79.69309		t-test	0.081428
6		68.22713		152.5673475	68.22713		NASH samples = 4 NASH F3 + 2 NASH F1 + 2 NASH F0 (total 8 samples)	
7		73.07209		149.6953309	73.07209			
8		77.7064			77.7064			
	t-test	0.000211		t-test	0.000167			
